# Hydrolysis-Engineered Robust Porous Micron Silicon Anode for High-Energy Lithium-Ion Batteries

**DOI:** 10.1007/s40820-025-01808-y

**Published:** 2025-06-13

**Authors:** Mili Liu, Jiangwen Liu, Yunqi Jia, Chen Li, Anwei Zhang, Renzong Hu, Jun Liu, Chengyun Wang, Longtao Ma, Liuzhang Ouyang

**Affiliations:** 1 School of Materials Science and Engineering, Guangdong Provincial Key Laboratory of Advanced Energy Storage Materials, South China University of Technology, Guangzhou, 510641 People’s Republic of China; 2 Guangdong Engineering Technology Research Center of Advanced Energy Storage Materials, Guangzhou, 510641 People’s Republic of China; 3 GAC Automotive Research & Development Center, Guangzhou, 511434 People’s Republic of China

**Keywords:** Micro-sized silicon anode, Pore structure, Functionalized SiO_*x*_/C interface, Long-term lithium-ion batteries

## Abstract

Micro-silicon (Si) anode that features high theoretical capacity and fine tap density is ideal for energy-dense lithium-ion batteries. However, the substantial localized mechanical strain caused by the large volume expansion often results in electrode disintegration and capacity loss. Herein, a microporous Si anode with the SiO_*x*_/C layer functionalized all-surface and high tap density (~ 0.65 g cm⁻^3^) is developed by the hydrolysis-driven strategy that avoids the common use of corrosive etchants and toxic siloxane reagents. The functionalized inner pore with superior structural stability can effectively alleviate the volume change and enhance the electrolyte contact. Simultaneously, the outer particle surface forms a continuous network that prevents electrolyte parasitic decomposition, disperses the interface stress of Si matrix and facilitates electron/ion transport. As a result, the micron-sized Si anode shows only ~ 9.94 GPa average stress at full lithiation state and delivers an impressive capacity of 901.1 mAh g⁻^1^ after 500 cycles at 1 A g⁻^1^. It also performs excellent rate performance of 1123.0 mAh g⁻^1^ at 5 A g⁻^1^ and 850.4 at 8 A g⁻^1^, far exceeding most of reported literatures. Furthermore, when paired with a commercial LiNi_0.8_Co_0.1_Mn_0.1_O_2_, the pouch cell demonstrates high capacity and desirable cyclic performance.

**Supplementary Information:**

The online version contains supplementary material available at 10.1007/s40820-025-01808-y.

## Highlights

- There is a novel “hydrolysis-driven synthesis” approach for the preparation of a dual-surface functionalized micron-sized Si anode with a SiO_*x*_/C layer.
- The functionalized inner pores and dual-functional SiO_*x*_/C layer synergistically alleviate volume change of Si lithiation, minimize stress concentration and improve electrochemical reaction kinetics.
- The optimized micron-Si anode performs impressive lifespan, excellent high rate capacity and outstanding stack cell volumetric energy density.

**Supplementary Information:**

The online version contains supplementary material available at 10.1007/s40820-025-01808-y.

## Graphical abstract

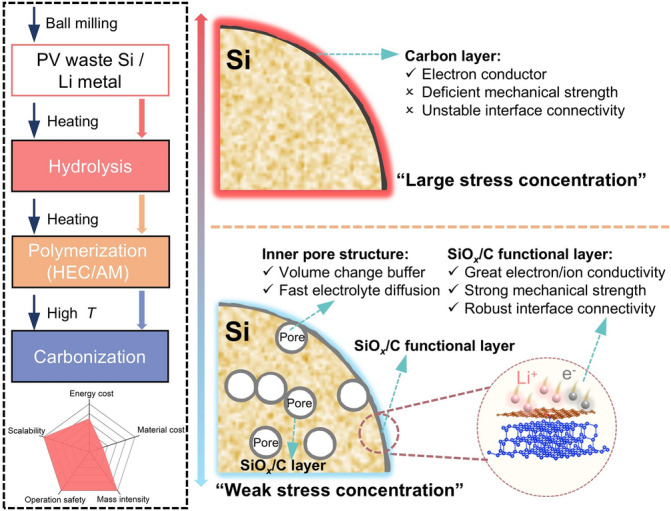

## Introduction

Silicon (Si) stands out as a highly promising anode material for next-generation lithium-ion batteries (LIBs) [1–3], thanks to its exceptional gravimetric capacity (3579 mAh g⁻^1^) [4–6], favorable equilibrium potential (~ 0.4 V vs. Li^+^/Li) and natural abundance [7, 8]. However, the practical application of Si anode has long been impeded by the electrode disintegration from large volume change (> 300%) during the (de)lithiation process, leading to sluggish reaction kinetics from the low intrinsic conductivity [9, 10], and the loss of Li^+^ inventory from the exacerbated solid electrolyte interphase (SEI) growth on the unstable interface [11–14]. To address above bottlenecks, porous nanostructured Si anodes have been widely explored to buffer the lithiation-induced mechanical stress for attenuating the material failure [15–18]. Nevertheless, nanostructures suffer from excessive specific surface area and low tap density (< 0.3 g cm⁻^3^), leading to high electrolyte consumption, poor coulombic efficiency and reduced volumetric energy density [19–22]. Besides, the fragility of nanostructures limits their ability to withstand high mechanical pressure of up to 80 MPa during common electrode calendaring. Moreover, the hazardous pore pre-plantation technologies that commonly use corrosive etchants like HF or high-concentrated alkaline solution also face limitations in terms of scalability, cost and environmental impact [23–25].

In comparison, micron-sized Si with smaller specific surface area and higher tap density gains attention as a practical and scalable alternative to the nanostructured silicon to improve volumetric energy density [26–28], whereas micron-sized Si anode still undergoes > 300% volume expansion, exacerbated SEI growth and poor electrical conductivity. Carbon layer coating has been commonly utilized to tackle the challenges associated with volume variation and electrical conductivity [29–32]. However, the absence of mechanical robustness of carbon layer fails to withstand repeated expansion and contraction of Si during lithiation/delithiation. The weak intermolecular forces of van der Waals and π – π stacking between carbon and Si matrix also fail to prevent the detachment of carbon network from Si particles and thus reduce electrode integrity [33–35]. Moreover, the concentrated strain always occurs at the edge of the Si surface, which accelerates the production of cracks for aggravating side reactions with the electrolyte and compromising structural stability [33, 36]. In addition, while a fully encapsulating carbon layer can improve interface stability [37], the carbon layer lacks enough Li^+^ conductivity to accelerate Li^+^ transport [36]. Therefore, as-designed carbon-based coating should not only enable excellent mechanical strength and electron conductivity but also impressive Li^+^ diffusion ability.

In this work, we proposed an innovative and sustainable hydrolysis-driven strategy to develop dual-surface functionalized microporous Si (p-mSi@SiO_*x*_/C), which is achieved *through* the “hydrolysis-polymerization-carbonization” process without requiring corrosive etchants and widely used toxic siloxane reagents. The designed p-mSi@SiO_*x*_/C electrode can exhibit enhanced electrochemical performance as follows: (1) The controlled inner pores can effectively alleviate the volume change of Si matrix during the lithiation/delithiation process and provide fast electrolyte diffusion pathways; (2) the inner and outer functional layers with robust connectivity with Si matrix not only mitigate stress concentrations at the Si interface but also facilitate Li⁺ ion transport; and (3) the partially SiO_*x*_ in dual-surface layer reacts to form lithium silicates during initial lithiation step, further stabilizing the electrode structure and acting as Li^+^ conductor to reduce Li^+^ diffusion barrier. Consequently, the as-prepared p-mSi@SiO_*x*_/C delivers a high initial reversible capacity of 1485.5 mAh g⁻^1^ and above 901.1 mAh g⁻^1^ at 1 A g⁻^1^ after 500 cycles, outperforming the Si (mSi, 308.5 mAh g⁻^1^ after 350 cycles) and carbon-coated mSi (mSi@C, 682.5 mAh g⁻^1^ after 350 cycles). When paired with a commercial high-loading LiNi_0.8_Co_0.1_Mn_0.1_O_2_ (NCM811) cathode, the pouch battery achieved 2.09 mAh cm⁻^2^ at 1.0 A g⁻^1^ over 100 cycles.

## Experimental Section

### Material Preparation

***Synthesis of MSi and mSi:*** The wasted photovoltaic Si chips were pulverized by ball milling with 300 rpm for 3 h to obtain micron-sized Si powder, while the balls–material ratio is 20:1. Then, as-obtained 2 g micron-sized Si powder and 0.008 g Li trips were ball milling with 450 r min^−1^ for 20 h to prepare the MSi, (The balls–material ratio is 50:1.). The mSi was synthesized by a similar ball milling method without the addition of Li trips.

***Synthesis of p-mSi@SiO***_***x***_***/C and mSi@C:*** Firstly, 0.2 g MSi powder was hydrolyzed in the pure water at 55 °C within 1 h. After that, 0.2 M acetic acid was added into the mixture to adjust the pH to 6.0. Then, a certain amount of hydroxyethyl cellulose and acrylamide (mass ratio:1/6, Table S1) was added to dispersion and stirred evenly at 55 °C for 3 h. The initiator and crosslinker were VA-044 and N, N'-Methylenebisacrylamide, respectively. After further stirring and drying at 80 °C, the precursor was calcinated at 250 °C for 1 h and 700 °C for 3 h in the Ar atmosphere to acquire p-mSi@SiO_*x*_/C. Meantime, the p-mSi@SiO_*x*_/C-3, p-mSi@SiO_*x*_/C-5, and p-mSi@SiO_*x*_/C-7 were prepared with a prolonged hydrolysis time of 3, 5, and 7 h, respectively, while other synthesis conditions remained unchanged. For comparison, the mSi@C was also prepared via a similar process without the hydrolysis process.

### Material Characterization

The XRD patterns of samples were analyzed by the powder diffractometer (PANalytical EMPYREAN) with CuKα radiation. The carbon content of samples was recorded using a thermal gravimetry analyzer (NETZSCH TG 209 F3 Tarsus). The Raman spectra were conducted using DXR2xi Raman microscope with a laser excitation wavelength of 633 nm. The specific surface area of samples was collected using N_2_ adsorption–desorption analysis (Quantachrome Autosorb iQ Station). The tap density of materials was measured using the tap density tester (Bettersize B1-301). The XPS spectra were tested by the X-ray photoelectron spectrometer (Thermo Fisher Scientific K-Alpha). The morphologies and structures were captured using SEM (Zeiss TESCAN GAIA 3 model 2016 UHR) and TEM (Talos F200X), respectively. EDS mapping results were tested by the Super-X EDS system. TOF–SIMS images and spectra were collected by using a TOF–SIMS spectrometer attached to the SEM. AFM characterizations were collected using a Bruker Dimension Icon. FTIR spectra of electrodes were collected by ATR-FTIR spectrometer (Nicolet IS50).

### Electrochemical Measurements

A homogenous slurry containing the active material (70 wt%), carboxymethyl cellulose sodium (Na-CMC, 10 wt%) and conductive carbon (19.2 wt% C45 + 0.8 wt% CNT) was well mixed in the deionized water. The slurry was further cast onto Cu foil and dried in a vacuum oven at 80 °C for 12 h, and the mass loading of active materials was calculated at about 0.6–0.8 mg cm⁻^2^. The 2025-type coin cells were assembled with lithium foils as counter electrode and 1 M LiPF_6_ in DEC/EC = 2:1 Vol% with 10%FEC as the electrolyte. The galvanostatic performance was conducted using a NEWARE battery tester with a voltage range of 0.01–1.0 V. The EIS and CV curves were collected by an electrochemical workstation (Gamry interface 5000P). For the pouch cell, the p-mSi@SiO_*x*_/C anode was prepared by mixing the p-mSi@SiO_*x*_/C, carboxymethyl cellulose and conductive carbon with a weight ratio of 90:5:5. Then, the pouch cell was fabricated using p-mSi@SiO_*x*_/C anode and the commercial NCM811 cathode (18.75 mg cm⁻^2^) as counter electrode with a N/P ratio of 1:1.14.

### Theoretical Calculations

The DFT calculations were executed using the Vienna Ab initio Simulation Package [38] (VASP). The van der Waals (vdW) correction was considered by applying the DFT-D3 approach [39, 40]. The projector-augmented plane-wave (PAW) pseudopotentials were employed, coupled with the generalized gradient approximation using the Perdew–Burke–Ernzerhof functional (GGA-PBE), to effectively describe the exchange–correlation function [41, 42]. As for geometry relaxation, the Brillouin zone was sampled with Gamma (Γ)- centered Monkhorst–Pack mesh with the K-mesh of 0.03 Å⁻^1^. Throughout this study, we employed a plane-wave cutoff energy of 520 eV and performed structural relaxation using the conjugate gradient algorithm until the residual force on each atom was reduced below 0.02 eV Å^−1^.

The stress distribution of the materials during the lithiation process was carried out by the finite element simulation based on the COMSOL Multiphysics 6.1 software. The particle model was designed with a particle radius of R_1_ = 2.5 μm, a coating shell thickness of 0.2 μm and a pore radius of R_2_ = 0.2 μm for better observation. A linear-elastic couple model was applied to replace the Li diffusion model to analyze the stress distribution, as shown in Eq. 1:

1 $$0 = \nabla \cdot S + F_{V}$$

where *S* is the stress tensor (Pa), and *F*_*V*_ is the volumetric stress (MPa). For simplicity and the purpose of investigating stress distribution and evolution upon lithiation, it was assumed that no interfacial separation occurred during the lithiation process.

## Results and Discussion

### Structural Stability Analysis and Material Design

**Fig. 1:**
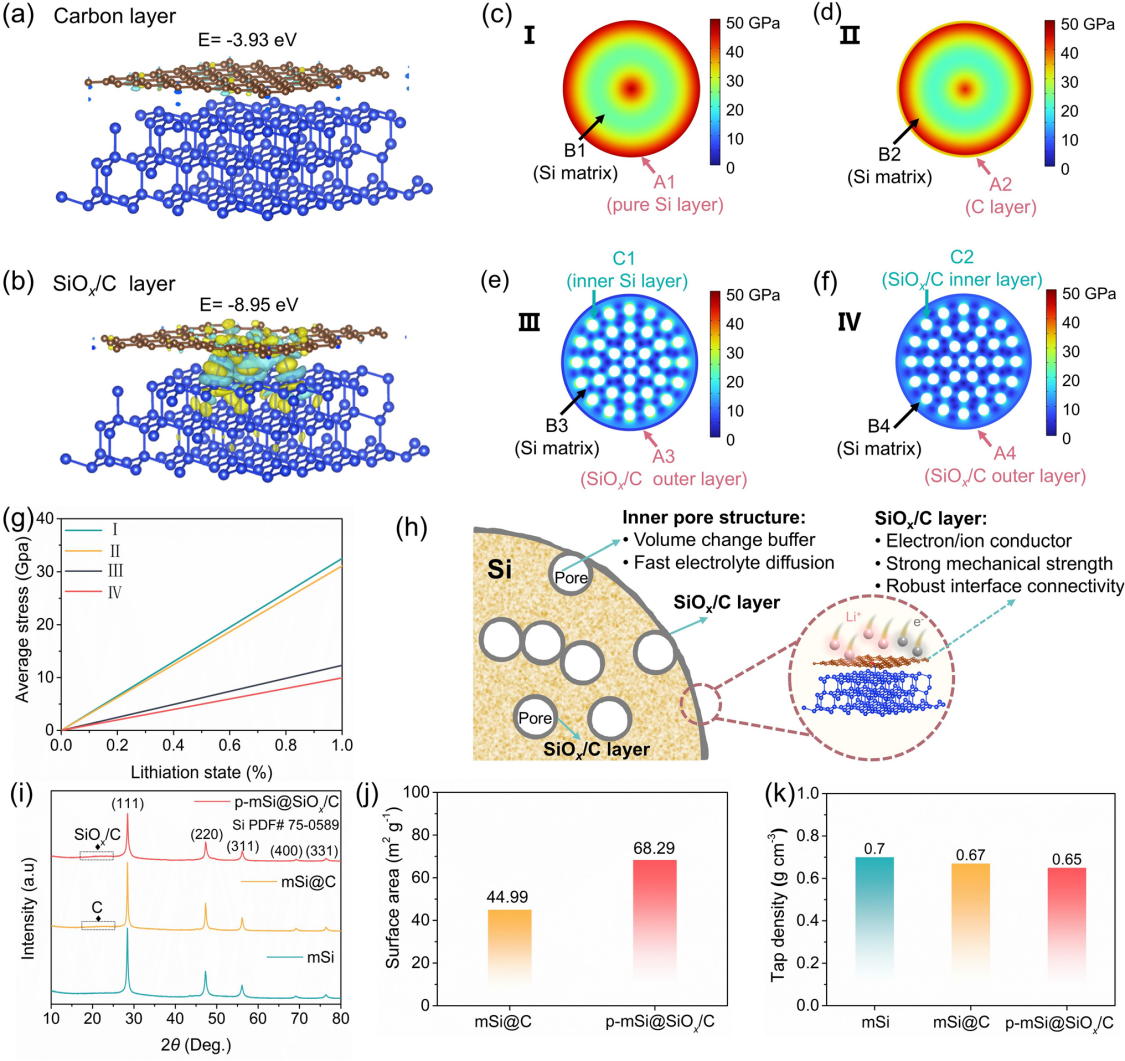
Structural simulation and design of p-mSi@SiO_*x*_/C. Energy for the **a** carbon and **b** SiO_*x*_/C layer bound to Si matrix. Stress distribution of **c** pure Si, **d** carbon-coated Si, **e** outer SiO_*x*_/C layer-coated porous Si and **f** both inner and outer SiO_*x*_/C layer-coated porous Si under full lithiation state. **g** Average stress distribution of four samples upon lithiation process. **h** Schematic diagram of the structural advantages of p-mSi@SiO_*x*_/C. **i** XRD patterns of mSi, mSi@C and p-mSi@SiO_*x*_/C. **j** Specific surface area of mSi@C and p-mSi@SiO_*x*_/C. **k** Tap density of mSi, mSi@C and p-mSi@SiO_*x*_/C

We begin by demonstrating the favorable interface stability of Si anode endowed by the SiO_*x*_/C coating layer through density functional theory (DFT) calculation. The higher binding energy (E = -8.95 eV) of Si-SiO_*x*_/C structure compared to Si–C (E = − 3.93 eV), as shown in Fig. 1a, b, suggests stronger interaction between Si matrix and SiO_*x*_/C layer, which is beneficial to mitigate stress from the repeated volume expansion/contraction of Si and thus maintain the structural integrity. Furthermore, the finite element simulation further reveals the stress distribution in the cross-sectional structure of various configurations: pure Si (I), carbon-coated Si (II), outer SiO_*x*_/C layer-coated porous Si (III, the surface of inner pore is uncoated), both inner and outer SiO_*x*_/C layer-coated porous Si (IV, both inner pore surface and outer particle surface of Si are coated), as depicted in Figs. 1c-g and S1-S5. Although the carbon layer in carbon-coated Si slightly reduces surface stress (full lithiation state: A2 ~ 41.52 GPa vs. A1 ~ 47.15 GPa) and provides limited overall improvement to the Si matrix (full lithiation state: B2 ~ 24.50 GPa vs. B1 ~ 28.11 GPa), the electrode still often leads to fractures during cycling. In sharp contrast, the dual designs of introducing a SiO_*x*_/C coating layer and creating inner pores within Si matrix significantly weaken the stress on the surface and matrix (full lithiation: A3 ~ 9.46 GPa/B3 ~ 7.98 GPa), demonstrating the improved fatigue property of the surface and matrix structures. Notably, with the inner pore surfaces also covered by the SiO_*x*_/C layer, the stress concentrated at pore edges is further relieved (full lithiation state: C2 ~ 13.44 GPa vs. C1 ~ 29.37 GPa), thereby reducing surface and matrix stress (full lithiation: A4 ~ 8.51 GPa/B4 ~ 6.34 GPa). Correspondingly, the both inner and outer SiO_*x*_/C layer-coated porous Si shows the lowest average stress upon the lithiation process than others (Fig. 1g, full lithiation: IV ~ 9.94 GPa < III ~ 12.33 GPa < II ~ 31.14 GPa < I ~ 32.54 GPa), resulting to the enhanced structural stability for restraining particle fracture caused by volume expansion. Meantime, the commonly used Na-CMC binder in the electrode forms a robust adhesion to the Si particles, enabling good electronic conductivity between active Si material and carbon black [43]. Once the Si particles experience a significantly higher stress concentration than the elastic modulus of Na-CMC binder (~ 12.01 GPa) during the lithiation process [44], the Na-CMC binder would cause a toughness fracture and structural failure, resulting to electron transport loss and crack propagation in the electrode [45]. Obviously, the both inner and outer SiO_*x*_/C layer-coated porous Si with the lowest surface stress achieves superior mechanical stability and structural toughness, which can effectively prevent the separation of electrode islands and preserve excellent electron transfer during the long-term cycling.

Building on these theoretical insights, we developed the micron-sized p-mSi@SiO_*x*_/C anode using a green “hydrolysis-organic polymerization-carbonization” method, which features enriched pores and a complete inner and outer surface of dual SiO_*x*_/C coating. As illustrated in Fig. S6, the modified Si (MSi) sourced from the photovoltaic waste reacts with water to form SiO_2_ sol, creating a hydrophilic Si matrix with abundant pores structure. The cost-efficiency hydroxyhexyl cellulose (HEC) with substantial hydroxide groups and the acrylamide (AM) with allyl structure are added to immobilize the SiO_2_ sol, forming an inorganic–organic interpenetrating network across both inner pores and outer particle surfaces of the Si matrix. Afterward, it is heated at a high temperature to obtain the final product. Figure S7 shows a fitting relationship between the conversion efficiency of MSi to SiO_2_ and hydrolytic reaction time, enabling the control of the SiO_2_ content in the SiO_*x*_/C layer to balance the lithiation capacity and structural stability. A series of samples with various hydrolytic time were synthesized, as described in detail in the Experimental Section, along with mSi and mSi@C for comparison. It is believed that the SiO_*x*_/C layer can form satisfied electron/Li^+^ conductive pathways and a firm protection layer with strong mechanical strength, and the pore structure acts as volume change buffer rooms and promotes electrolyte diffusion (Fig. 1h). Moreover, we have summarized three advanced Si anode preparation methods and compared their scalability and economic viability with our work (Fig. S8). The analysis was conducted according to their energy and material flows. While all methods demonstrate promising potential for scale-up and practical implementation, critical differences emerge in specific operational aspects. As shown in Fig. S8a, b, the magnesiothermic reduction-based approach (scenario 1) proposed by the Luan et al. [46] requires two high-temperature heating treatments coupled with a CVD process, resulting to huge energy consumption. Additionally, its HCl etching process generates excess MgCl_2_ by-products, compromising material cost and mass intensity. The AgNO_3_/HF etching-based method (scenario 2) developed by Li et al. [47] achieves significant material cost advantages through the use of recycled silicon and low-cost organic coating agents, yet faces operational safety challenges due to its hazardous HF acid processing step (Fig. S8c, d). The covalent organic framework coating-based strategy (scenario 3) designed by Fan et al. [48] demonstrates great promises in energy cost, mass intensity and operation safety, but its commercial viability is constrained by using expensive nanosilicon and high-cost organic ligands (Fig. S8e, f). In comparison, our “hydrolysis-polymerization-carbonization” method displays notable advances in material cost, mass intensity and operation safety (Fig. S8g, h). However, the energy consumption and the throughput per production batch present potential challenges for optimization, as shown in the cost analysis of production flow (Table S2). Moreover, the inert atmosphere storage of the milled precursor would introduce additional production costs.

X-ray diffraction (XRD) patterns (Fig. 1i) show the peaks corresponding to Si (111), (220), (311), (222), and amorphous carbon/SiO_*x*_/C (~ 22°) of the mSi, mSi@C and p-mSi@SiO_*x*_/C, where a set of diffraction peaks belonged to increasing SiO_2_ content in the SiO_*x*_/C layer through prolonging hydrolysis time enhances the SiO_*x*_/C peak while reducing Si peak intensity (Fig. S9). The thermogravimetric analysis (TGA) suggests a consistent carbon content of 20 wt%–22 wt% across mSi@C and all p-mSi@SiO_*x*_/C samples (Fig. S10). The Raman spectra reveal a dominant Si–Si vibration peak located at 516 cm⁻^1^, characteristic of crystalline Si (Fig. S11), with the consistent graphitized nature across all samples, as indicated by similar D/G band intensity ratio (*I*_*D*_*/I*_*G*_). The Brunauer–Emmett–Teller (BET) analysis (Fig. S12a) shows a specific surface area of 68.29 m^2^ g⁻^1^ for p-mSi@SiO_*x*_/C, slightly higher than that of mSi@C (44.99 m^2^ g⁻^1^) (Fig. 1j). The p-mSi@SiO_*x*_/C also exhibits a higher ratio for the pore size in 5–8 nm, which is assigned to the inner nanopore structure (Fig. S12b). In addition, with the prolonging of hydrolysis time, the pore size of products gradually increases and the total pore volume of mSi, p-mSi@SiO_*x*_/C, p-mSi@SiO_*x*_/C-3, p-mSi@SiO_*x*_/C-5 and p-mSi@SiO_*x*_/C-7 is 0.042, 0.069, 0.079, 0.209 and 0.243 cm^3^ g⁻^1^, respectively, indicating the enhancement of porosity for the Si matrix (Fig. S12b, c). These pore structures are beneficial to alleviate the volume expansion of Si lithiation. Encouragingly, as shown in Fig. 1k, p-mSi@SiO_*x*_/C achieves a decent tap density of 0.65 g cm⁻^3^, only slightly lower than that of mSi (0.7 g cm⁻^3^) and mSi@C (0.67 g cm⁻^3^). The combination of high tap density and suitable inner pore structure supports high energy density while minimizing stress concentration for better electrode–electrolyte interfacial stability.

**Fig. 2:**
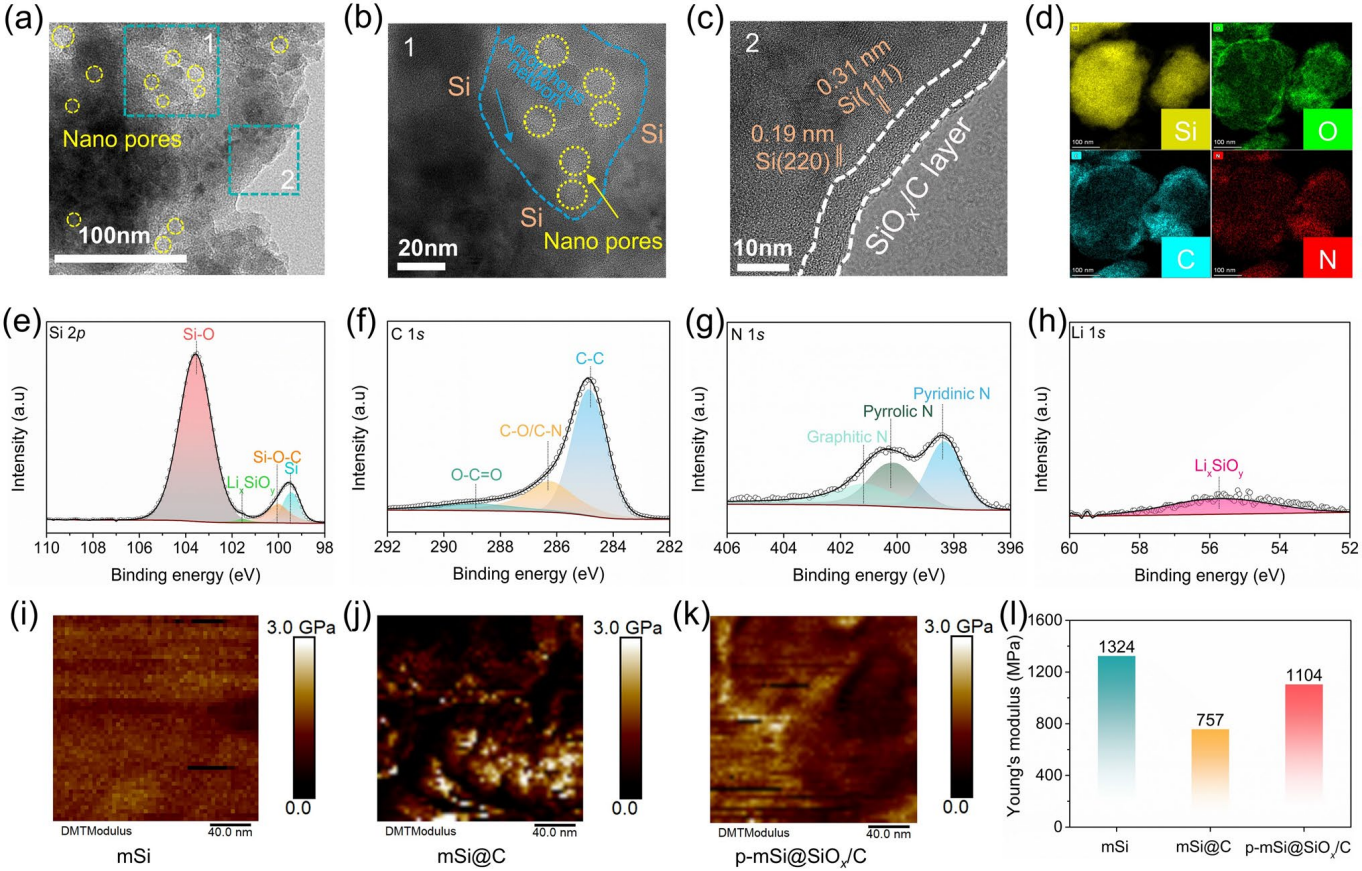
Characterization of as-prepared materials. **a** TEM, **b, c** HRTEM and **d** EDS mapping images of p-mSi@SiO_*x*_/C. **e–h** High-resolution Si 2*p*, C 1*s*, N 1*s* and Li 1*s* spectra of p-mSi@SiO_*x*_/C. **i-k** AFM test results and corresponding **l** Young’s modulus of mSi, mSi@C and p-mSi@SiO_*x*_/C

The morphology and microstructure of the as-prepared materials are characterized by scanning electron microscopy (SEM) and transmission electron microscopy (TEM). Compared to the mSi with a smooth surface, the mSi@C depicts a coarse and dense surface due to the adhesion of pyrolytic carbon derived from HEC/AM (Fig. S13a, b). The p-mSi@SiO_*x*_/C exhibits a similarly coarse surface but with a large number of nanopores (Fig. S13c, d). The edges of nanopores are uniformly covered by the SiO_*x*_/C layer to form a conductive network rather than fully filling the pores. The size of inner nanopores is about 5–10 nm, and the thickness of SiO_*x*_/C layer is approximately 7.3 nm (Fig. 2a-c). The energy-dispersive spectroscopy (EDS) mapping of p-mSi@SiO_*x*_/C confirms the uniform distribution of Si, O, C and N elements (Fig. 2d). The surface chemical compositions of p-mSi@SiO_*x*_/C are reflected by the X-ray photoelectron spectroscopy (XPS) spectra. The Si 2*p* spectrum can be divided into four peaks including Si–O (103.6 eV), Li_*x*_SiO_*y*_ (101.5 eV), Si–O–C (100.0 eV) and Si^0^ (99.4 eV) [49, 50], in which the Si–O and Si–O–C bonds highlight the structural features of SiO_*x*_/C layer, including SiO_*x*_ composition and Si–O–C network (Fig. 2e). The content of SiO_*x*_ within the SiO_*x*_/C layer increases with longer hydrolysis times (Fig. S14). The presence of Li_*x*_SiO_*y*_ species is attributed to the reaction product between the Li sources and SiO_2_ sol, further affirmed by the Li 1*s* spectrum with the peak at 55.6 eV (Fig. 2h) [51, 52]. In addition, the C 1*s* and N 1*s* spectra show the characteristic peaks of C-N species (Fig. 2f, g), indicating the nitrogen-doped carbon structure that enhances charge transfer in the electrode [47, 53, 54].

The mechanical properties of the surface structure are evaluated using the atomic force microscope (AFM) to measure Young’s modulus. As shown in Fig. 2i–l, the coating modification imparts a lower Young’s modulus to modified Si compared to mSi, which enhances toughness and reduces stress concentration and thereby improving the restoring ability of cracks. However, mSi@C presents an excessively low Young’s modulus of 757 MPa due to the soft carbon layer, which can detach from the Si matrix during long-term cycling. In comparison, p-mSi@SiO_*x*_/C achieves a moderate modulus of 1104 MPa, balancing hardness and softness [55]. This trade-off ensures structural integrity and reduces mechanical exfoliation between SiO_*x*_/C coating and the inner Si core during operation, making the p-mSi@SiO_*x*_/C highly robust.

### Electrochemical Performance

**Fig. 3:**
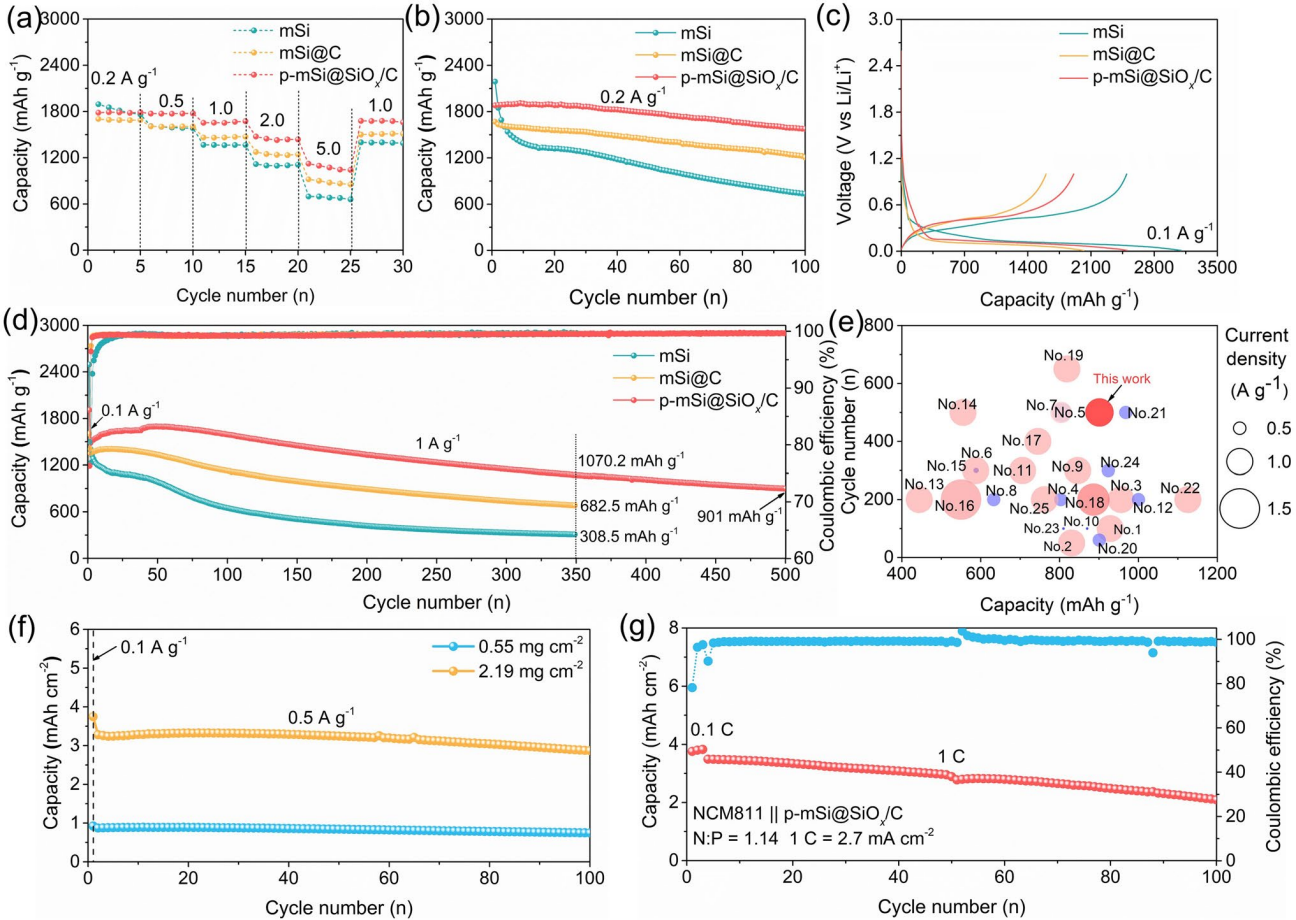
Electrochemical performance. **a** Rate capabilities of mSi, mSi@C and p-mSi@SiO_*x*_/C. **b** Cyclic stability of mSi, mSi@C and p-mSi@SiO_*x*_/C at 0.2 A g⁻^1^. **c** The first charge/discharge profiles of mSi, mSi@C and p-mSi@SiO_*x*_/C at 0.1 A g⁻^1^. **d** Cyclic stability of mSi, mSi@C and p-mSi@SiO_*x*_/C at 1.0 A g⁻^1^. **e** Comprehensive comparison of p-mSi@SiO_*x*_/C with reported advanced Si anodes. **f** Cyclic stability of p-mSi@SiO_*x*_/C at different mass loading. **g** Cyclic performance of p-mSi@SiO_*x*_/C in a pouch cell with commercial NCM811 cathode

The electrochemical performance of p-mSi@SiO_*x*_/C anode is evaluated using Li metal as the counter electrode within a discharge–charge voltage window of 0.01–1.0 V. The rate capability tests at current densities ranging from 0.2 to 5 A g^–1^ demonstrate the excellent electrochemical reaction kinetics of the p-mSi@SiO_*x*_/C, which delivers high reversible charge capacities of 1783.6, 1772.3, 1653.1, 1474.6 and 1123.0 mAh g^–1^ at current densities of 0.2, 0.5, 1.0, 2.0 and 5.0 A g^–1^, respectively, remarkably outperforming mSi and mSi@C (Fig. 3a). When the current density returns to 1 A g^–1^, the capacity of p-mSi@SiO_*x*_/C restores to 1677.5 mAh g^–1^, underscoring its outstanding rate capability and potential for practical application. In addition, Fig. S15 exhibits the discharge–charge profiles and corresponding average lithiation/delithiation voltage of various electrodes at different current densities. All these electrodes show good stability during the rate-tests process, as demonstrated by the similar lithiation/delithiation behaviors at each current condition (Fig. S15a-c). However, besides the Li storage capacity, the p-mSi@SiO_*x*_/C displays higher average lithiation voltages than mSi and mSi@C across high current densities, contributing to reduced lithium plating risk for the fast charging applications (Fig. S15d). Moreover, the lower average delithiation voltage achieved by the p-mSi@SiO_*x*_/C than the mSi@C is beneficial to enable higher energy density of the full cell (Fig. S15e). Therefore, the stack energy density (*U*_*R*_), a critical parameter for the practical application of anode material, is calculated through the cell model of Obrovac et al. [56] (Eq. 2):

2 $${U}_{R}=\frac{\left(58327.5 Ah {L}^{-1}\right)}{70+110*\left(1+\frac{583.275 Ah {L}^{-1} }{{Q}^{-}}\right)}\left(3.9V-{V}_{ave}^{-}\right)$$

where the ${Q}^{-}$ and ${V}_{ave}^{-}$ represent the reversible volumetric capacity (Ah L⁻^1^) and average delithiation voltage (V) of the anode, respectively. As shown in Fig. S15f and Table S3, the p-mSi@SiO_*x*_/C obtains excellent stack energy densities at different current densities from 0.2 to 5 A g⁻^1^. Particularly at 5 A g⁻^1^, the stack energy density of p-mSi@SiO_*x*_/C maintains 896.4 Wh L⁻^1^, much superior to that of mSi (821.2 Wh L⁻^1^) and mSi@C (850.6 Wh L⁻^1^), also higher than the theoretical energy density of commercial graphite (~ 726 Wh L⁻^1^). Furthermore, we also assess the rate performance of p-mSi@SiO_*x*_/C at a wider current density range from 0.2 to 8 A g⁻^1^ (Fig. S16). Even at a higher rate condition of 8 A g⁻^1^, the p-mSi@SiO_*x*_/C still delivers an advanced reversible capacity of 850.4 mAh g⁻^1^ and an impressive stack energy density of 835.3 Wh L⁻^1^.

Moreover, the cyclic performance is measured under various current densities. As presented in Fig. 3b, the p-mSi@SiO_*x*_/C displays a high capacity of 1576.4 mAh g⁻^1^ after 100 cycles at the current density of 0.2 A g⁻^1^, far surpassing 733.7 and 1208.1 mAh g⁻^1^ achieved by mSi and mSi@C, respectively. Under 1 A g⁻^1^ cycling conditions, the cells were initially cycled at 0.1 A g⁻^1^ for one activation cycle prior to long-term cycling. As shown in Fig. 3c, the p-mSi@SiO_*x*_/C delivers 1st discharge/charge capacities of 2502.1/1909.0 mAh g⁻^1^ with a 76.3% initial coulombic efficiency. Moreover, the p-mSi@SiO_*x*_/C shows desirable long-term cyclic stability and impressive electrochemical reactivity at 1 A g⁻^1^. It achieves an initial reversible charge capacity of 1485.5 mAh g⁻^1^ after the first activation cycle, surpassing that of the mSi@C (~ 1349.9 mAh g⁻^1^) and mSi (1404.7 mAh g⁻^1^), and can gradually raise to 1697.3 mAh g⁻^1^ due to the deeper activation of electrode material. Furthermore, the p-mSi@SiO_*x*_/C retains a high reversible capacity of 1070.2 mAh g⁻^1^ after 350 cycles, corresponding to a capacity retention of 63.0% (compared to the highest capacity). In sharp contrast, the capacities of mSi and mSi@C decline rapidly to 308.5 mAh g⁻^1^/22.0% and 682.5 mAh g⁻^1^/48.5%, respectively. Moreover, the p-mSi@SiO_*x*_/C can still deliver a high reversible capacity of 901.1 mAh g^−1^ after 500 cycles. Further studies (Fig. S17) show that increasing SiO_*x*_ content in the SiO_*x*_/C layer enhances cyclic stability at the cost of a slight reduction in reversible capacity. We further comprehensively compare the developed p-mSi@SiO_*x*_/C anode with other reported micron-Si-based anodes, and it shows great advancements in terms of specific capacity, rate capability and cycling life (Fig. 3e and Table S4). The practicability of p-mSi@SiO_*x*_/C anode is further assessed under high mass loading. The electrode with mass loading of 0.55 and 2.19 mg cm⁻^2^ delivers stable cyclic ability with areal capacity of 0.75 and 2.87 mAh cm⁻^2^ at 0.5 A g⁻^1^ after 100 cycles (Fig. 3f). A pouch cell is assembled by using the p-mSi@SiO_*x*_/C anode paired with a commercial NCM811 (high mass loading = 18.75 mg cm⁻^2^) cathode. The pouch cell displays an excellent initial capacity of 3.49 mAh cm⁻^2^ and maintains 2.09 mAh cm⁻^2^ over 100 cycles at 1C (1C = 2.7 mAh cm⁻^2^) (Fig. 3g), showcasing its promise for practical applications.

### Electrochemical Reaction Kinetics and Electrode Structure Evolution

**Fig. 4:**
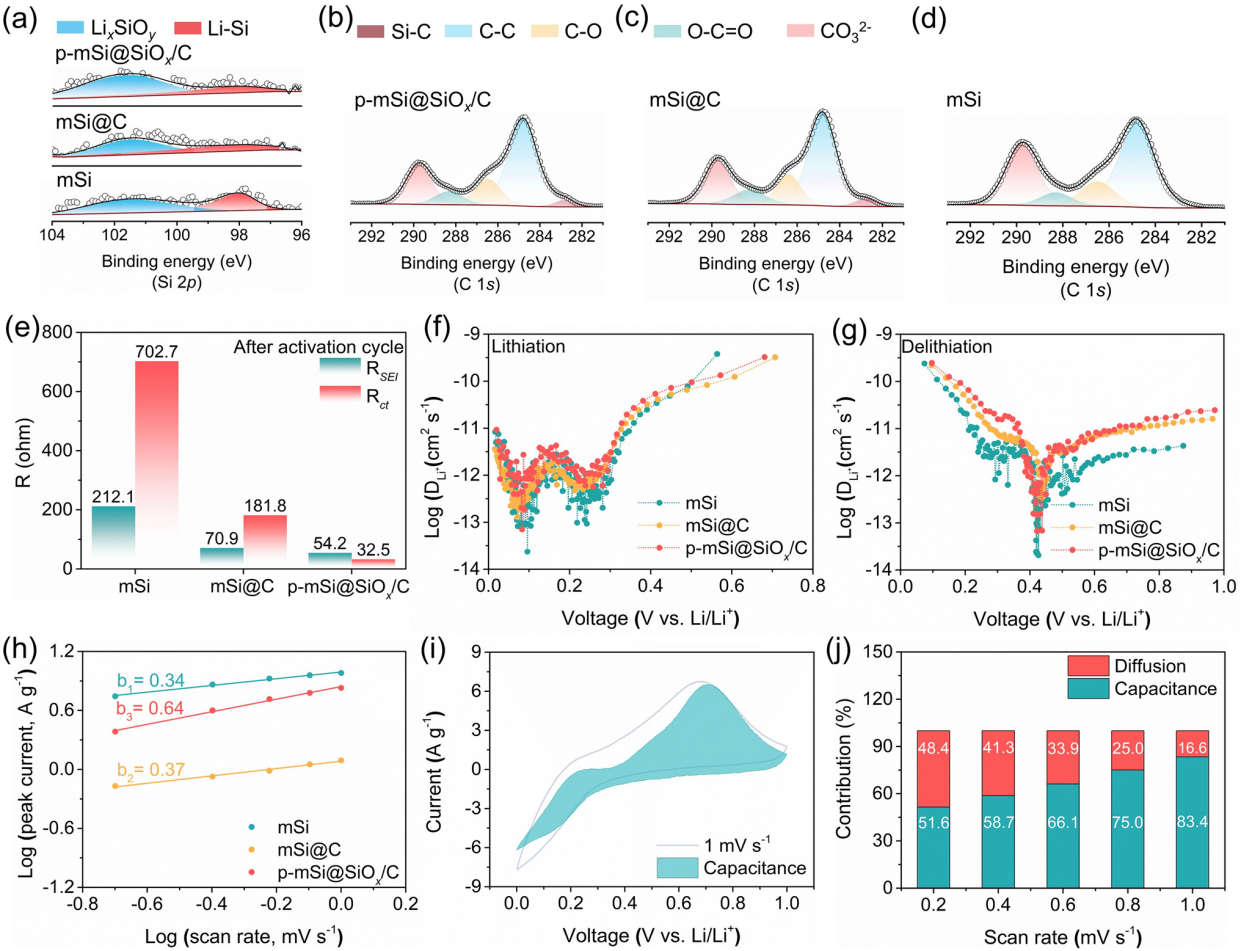
Electrochemical behavior investigations. **a** High-resolution Si 2*p* spectra of mSi, mSi@C and p-mSi@SiO_*x*_/C electrodes after the activation cycle. High-resolution C1*s* spectra of **b** mSi, **c** mSi@C and **d** p-mSi@SiO_*x*_/C electrodes after the activation cycles. **e** Resistance fitting values from the Nyquist plots for mSi, mSi@C and p-mSi@SiO_*x*_/C after the activation cycle. Li^+^ diffusion coefficient of **f** lithiation and **g** delithiation process for mSi, mSi@C and p-mSi@SiO_*x*_/C. **h** Plots of log (peak current) as a function of log (scan rate) to determine *b* values for mSi, mSi@C and p-mSi@SiO_*x*_/C. **i** Contribution of capacitance of p-mSi@SiO_*x*_/C at a scan rate of 1 mV s⁻^1^. **j** Percentages of diffusion and capacitance contributions of p-mSi@SiO_*x*_/C at different scanning rates

Aiming to better understand the effect of SiO_*x*_/C coating layer on the Li^+^ transfer and interface structure stability, the surface chemical environment of the activated electrodes is carried out by the XPS measurements. As shown in Fig. 4a, the activated mSi and mSi@C possess two Si 2*p* peaks of Li_*x*_SiO_*y*_ and Li-Si, which should be ascribed to the lithiation of native oxide layer and Si matrix. Compared with them, the activated p-mSi@SiO_*x*_/C exhibits a higher integral area and proportion for the Li_*x*_SiO_*y*_ peak, dominantly attributing to the lithiation of SiO_*x*_ in SiO_*x*_/C and thus leading to the formation of outperforming and stable Li^+^ diffusion pathways to the enfolded Si. Integrating with the intrinsic electron conductivity of carbon composition, the ion/electron dual conductor with outstanding kinetics is achieved by the SiO_*x*_/C layer. Moreover, the C 1*s* spectra results demonstrate that the p-mSi@SiO_*x*_/C shows the lowest C–O, C = O and O–C = O peaks area and ratio compared to the mSi and mSi@C, further confirming the weakest carbonates solvents decomposition (Fig. 4b–d). It indicates that the presence of SiO_*x*_/C is also conducive to the construction of a stable SEI structure and ensures lower interface resistance. The in situ Raman was further conducted to deeply investigate the modulation effect of the SiO_*x*_/C layer on the SEI formation during the first lithiation process. As shown in Fig. S18, for both mSi and p-mSi@SiO_*x*_/C, the peaks belonged to the EC (at ~ 970, ~ 892, and 715 cm⁻^1^), Li^+^-EC (at ~ 902 and ~ 727 cm⁻^1^) and LiPF_6_ (~ 740 cm⁻^1^) are detected at open circuit potential (OCP). During the main formation process of SEI film, the p-mSi@SiO_*x*_/C exhibits substantially weaker non-faradaic electrolyte adsorption and electrolyte reduction consumption compared to the mSi, which leads to an inorganic-rich SEI chemistry. Moreover, it is noteworthy that at the lithiation stage of Si, both EC, Li^+^-EC and LiPF_6_ configurations in the electrode/electrolyte interface of p-mSi@SiO_*x*_/C possess stronger signal intensity than the mSi, also demonstrating the superior interfacial stabilization of p-mSi@SiO_*x*_/C that effectively prevents continuous electrolyte decomposition. Therefore, the FTIR spectra peaks of O–C = O, CH_2_, C–O, P-O-C and P–F structures show lower intensity in the lithiated p-mSi@SiO_*x*_/C electrode (Fig. S19), further confirming the reduced content of organic species in the SEI film, which is conducive to improving chemical stability and mechanical strength of interfacial structure.

The electrochemical characteristics and Li diffusion kinetics of the as-prepared anodes are evaluated by electrochemical impedance spectroscopies (EIS), galvanostatic intermittent titration technique (GITT) and cyclic voltammetry (CV). As shown in Fig. 4e and Fig. S20, the activated p-mSi@SiO_*x*_/C anode displays lower resistance for the lithium transport through the SEI film (R_*SEI*_ ~ 54.2 Ω) and the charge transfer (R_*ct*_ ~ 32.5 Ω) than those of mSi (~ 212.1 Ω and 702.7 Ω) and mSi@C (~ 70.9 Ω and 181.8 Ω). Meantime, the Li^+^ diffusion coefficient (D_Li_^+^) calculated from the GITT measurement indicates that the p-mSi@SiO_*x*_/C exhibits significantly higher D_Li_^+^ values compared to the mSi and mSi@C (Figs. S21 and 4f, g). It further manifests that the p-mSi@SiO_*x*_/C shows lower diffusion barriers and enhanced reaction kinetics, which can be attributed to the conversion of SiO_*x*_ in SiO_*x*_/C layer to lithium silicate with high Li^+^ conductivity during the first lithiation process. Moreover, the CV measurements are conducted at various scanning rates from 0.2 to 1.0 mV s⁻^1^ to distinguish the lithium storage behavior, particularly the surface pseudocapacitive effect (Figs. 4h and S22). The peak current is logarithmically fitted against the scanning rate based on the function of $i=a{v}^{b}$, for identifying the b value. The *b* = 0.5 or 1 represents the ideal diffusion-controlled and surface pseudocapacitive-dominant process, respectively. The p-mSi@SiO_*x*_/C demonstrates a higher *b* value of 0.64 than that of mSi (~ 0.34) and mSi@C (~ 0.37), demonstrating that the lithium storage is synergistically determined by the diffusion-controlled and surface pseudocapacitive-dominant process. In addition, the contribution of diffusion-controlled (${k}_{1}{v}^{1/2}$) and surface pseudocapacitive-dominant process (${k}_{2}v$) can be quantitated *through* the equation of $i\left(V\right)= {k}_{1}{v}^{1/2}+{k}_{2}v$. The proportion of pseudocapacitive increases with enhanced scan rate, and it accounts for 83.4% of the total storage capacity at a scan rate of 1 mV s⁻^1^ (Fig. 4i, j). This improved pseudocapacitive behavior is critical for achieving excellent rate performance.

**Fig. 5:**
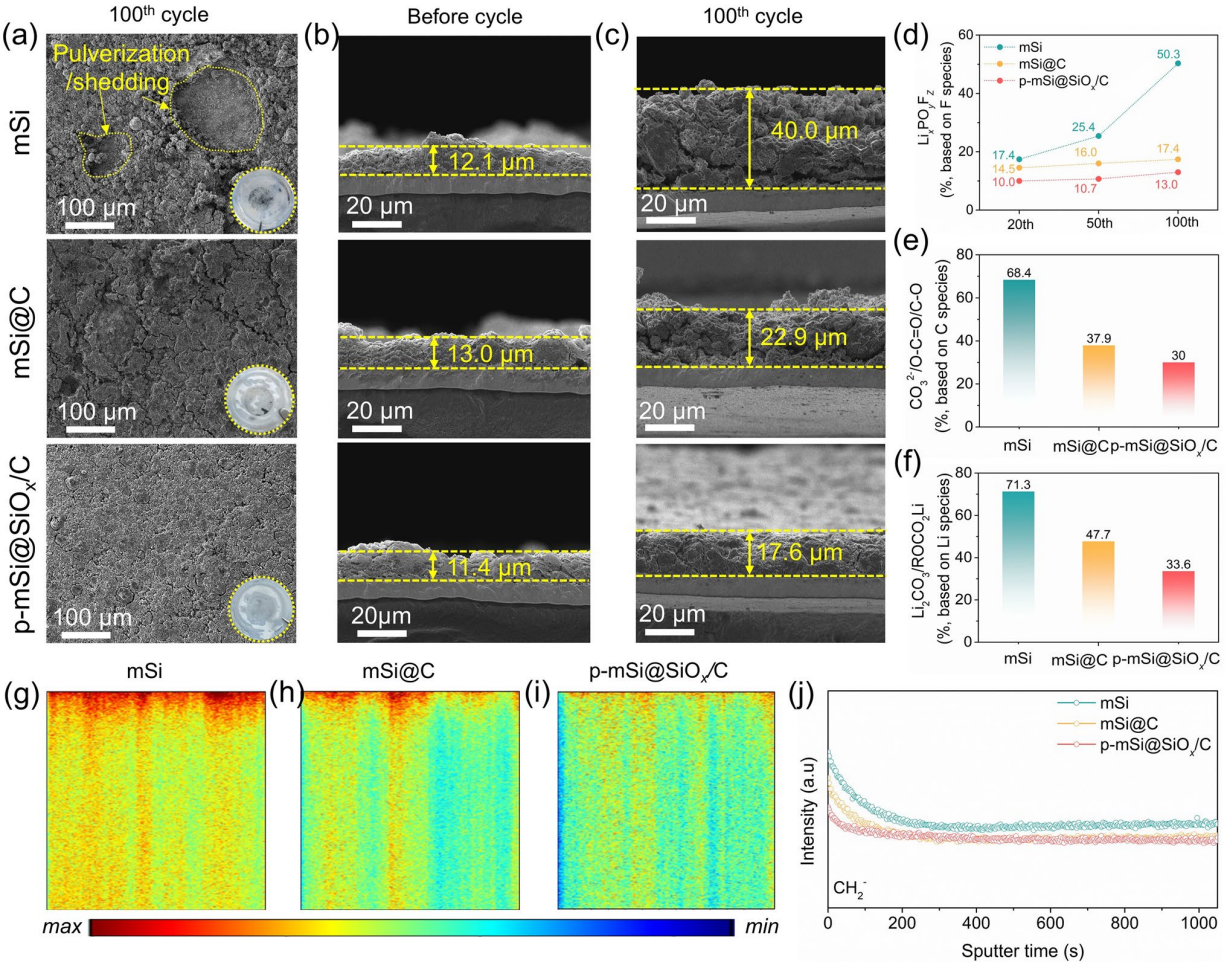
Analysis of cycled electrodes. **a** Surface and **b, c** cross-sectional SEM images of mSi, mSi@C and p-mSi@SiO_*x*_/C before and after 100 cycles at 1 A g⁻^1^; the insets belong to the separators after cycling. **d** Ratio analysis of Li_*x*_PO_*y*_F_*z*_ species based on F 1*s* spectra for mSi, mSi@C and p-mSi@SiO_*x*_/C electrodes within different cycles. Ratio analysis of **e** CO_3_^2−^/O-C = O/C-O and **f** Li_2_CO_3_/ROCO_2_Li for the surface of cycled electrodes. The vertical TOF–SIMS mappings of CH_2_^−^ cluster for the cycled **g** mSi, **h** mSi@C and **i** p-mSi@SiO_*x*_/C electrodes and **j** corresponding profiles

To evaluate the structural stability of p-mSi@SiO_*x*_/C, cells cycled at 1.0 A g⁻^1^ after 100 cycles are disassembled to observe morphological changes (Fig. 5a–c). Compared to the pristine electrode with a compact surface (Fig. S23), the cycled mSi and mSi@C electrodes exhibit severe pulverization, shedding of active materials and the formation of large cracks. Meantime, the expansion rates of the electrode thickness for mSi and mSi@C are 230.6% and 76.2%, respectively, manifesting that a simple carbon layer coating is insufficient to accommodate the volume expansion and maintain the interfacial stability, which results to structural breakdown and excessive SEI formation via electrolyte consumption, and rapid capacity fading. In sharp comparison, the cycled p-mSi@SiO_*x*_/C shows minimal surface cracking and significantly less active material deposition on the separator. The thickness expansion ratio is much lower at 45.6%, highlighting the advancement of SiO_*x*_/C layer for mitigating strain and enhancing interfacial stability. Moreover, the cycled mSi and mSi@C electrodes appear large cracks and obvious separation between active materials and Cu foil due to the huge lithiation stress and volume contraction effect, resulting to severe structural collapse and electronic contact loss. Conversely, only tiny cracks form in the cycled p-mSi@SiO_*x*_/C electrode, demonstrating a better structural failure resistance than the mSi and mSi@C electrodes. Additionally, as shown in Fig. S24, the cycled p-mSi@SiO_*x*_/C particle demonstrates fine structural integrity without visible cracks. Moreover, an amorphous layer of approximately 10–15 nm encapsulates the Si matrix, which could be identified as the mixture of SEI and SiO_*x*_/C layer based on the uniform distribution of Si, O, F, C and N elements, further confirming the structural stability of SiO_*x*_/C layer.

In addition, to further reveal the interfacial stability and SEI evolution, the XPS was conducted on mSi, mSi@C and p-mSi@SiO_*x*_/C electrodes within different cycles. The F species of the SEI film across the anode belongs to LiF and Li_*x*_PO_*y*_F_*z*_ [27, 57–59]. The p-mSi@SiO_*x*_/C electrode shows higher F signals and lower integral proportion of Li_*x*_PO_*y*_F_*z*_ signal than mSi and mSi@C at various cycles, demonstrating stronger mechanical property of SEI film and reduced electrolyte decomposition chemistry in the interface of electrode (Figs. 5d and S25). Moreover, the C 1*s* peaks associated with the C–O, O–C = O and CO_3_^2−^ are detected for the organic compositions, originating from decomposed carbonate solvents of electrolyte [60–63]. The 100th-cycled p-mSi@SiO_*x*_/C electrode shows the lowest total intensity and integral area and proportion (Figs. S26a and 5e), demonstrating a remarkably reduced solvent decomposition. The Li 1*s* peaks associated with Li_2_CO_3_ and ROCO_2_Li show the lowest ratio in the 100th-cycled p-mSi@SiO_*x*_/C (Figs. S26b and 5f), further confirming minimized parasitic reaction of organic solvents on the p-mSi@SiO_*x*_/C surface [64]. The TOF–SIMS analysis further gains more insights into the local electrolyte decomposition in the electrodes after 100 cycles. The TOF–SIMS depth profiles confirm the composition of fragments from the specimen during the sputtering process. As shown in Figs. 5g–j and S27- S28, the CH_2_^−^, C_2_H_2_O^−^ and PO^−^ species are much less generated in the surface and inner of cycled p-mSi@SiO_*x*_/C, again demonstrating an obviously alleviated electrolyte decomposition and strengthened electrode stability. Correspondingly, as shown in Fig. S29, the cycled p-mSi@SiO_*x*_/C exhibits lower resistance of R_*SEI*_ (~ 8.0 Ω) and R_*ct*_ (~ 6.9 Ω), compared to the cycled mSi (~ 49.2 and 35.3 Ω) and cycled mSi@C (~ 11.2 and 13.2 Ω). Therefore, the SiO_*x*_/C buffer layer in p-mSi@SiO_*x*_/C effectively mitigates the pulverization and cracking of the Si matrix, minimizes parasitic reactions and promotes the formation of a stable SEI with robust mechanical properties, thereby enabling outperformed cycling performance.

## Conclusions

In summary, a high-tap-density porous micron-sized Si anode with SiO_*x*_/C layer functionalized both inner pore structure and outer particle surface is developed by an eco-friendly “hydrolysis-polymerization-carbonization” strategy without relying on hazardous agents. The SiO_*x*_/C layer-functionalized inner pore structure provides a “breathing room” to accommodate the huge volume expansion, while the SiO_*x*_/C layer distributes reduced strain uniformly across all surfaces to prevent stress concentration and encourages electron/Li^+^ transfer during lithiation and delithiation of silicon anode. With the synergistic effect of the inner pore structure and SiO_*x*_/C functional layer, the designed micron-Si anode displays a super-low Si anode thickness expansion and remarkable electrochemical performance in terms of high reversible charge capacity (1485.5 mAh g⁻^1^ at 1 A g⁻^1^), rate capability (1123.0 mAh g⁻^1^ at 5 A g⁻^1^ and 850.4 mAh g^−1^ at 8 A g⁻^1^) and long-term cyclic stability (~ 901.1 mAh g⁻^1^ at 1 A g⁻^1^ after 500 cycles). This study provides insights into the role of inner pore structures and dual-functional surface engineering that can be harmonized to address the inherent challenges of silicon anodes, specifically their large volume changes.

## Supplementary Information

Below is the link to the electronic supplementary material.

Supplementary file1 (DOCX 33862 KB)
