# Supplementary material for: Hydrolysis-Engineered Robust Porous Micron Silicon Anode for High-Energy Lithium-Ion Batteries
Source: Nanomicro Lett. 2025 Jun 13;17:297. doi: 10.1007/s40820-025-01808-y (PMC12165945; doi:10.1007/s40820-025-01808-y)
Supplement: Supplementary file 1 — Supplementary file1 (DOCX 33862 KB) [file 40820_2025_1808_MOESM1_ESM.docx]

Supporting Information for

**Hydrolysis-Engineered Robust Porous Micron Silicon Anode for High-Energy Lithium-Ion Batteries**

Mili Liu^1^, Jiangwen Liu^1^, Yunqi Jia^1^, Chen Li^1^, Anwei Zhang^3^, Renzong Hu^1^, Jun Liu^1^, Chengyun Wang^3^, Longtao Ma^1^*, Liuzhang Ouyang^1,2^*

^1^ School of Materials Science and Engineering, Guangdong Provincial Key Laboratory of Advanced Energy Storage Materials, South China University of Technology, Guangzhou 510641, P. R. China

^2^ Guangdong Engineering Technology Research Center of Advanced Energy Storage Materials, Guangzhou 510641, P. R. China

^3^ GAC Automotive Research & Development Center, Guangzhou 511434, P. R. China

*Corresponding authors. E-mail: [longtaoma@scut.edu.cn](mailto:longtaoma@scut.edu.cn) (Longtao Ma); [meouyang@scut.edu.cn](mailto:meouyang@scut.edu.cn) (Liuzhang Ouyang)

**Supplementary Figures and Tables**


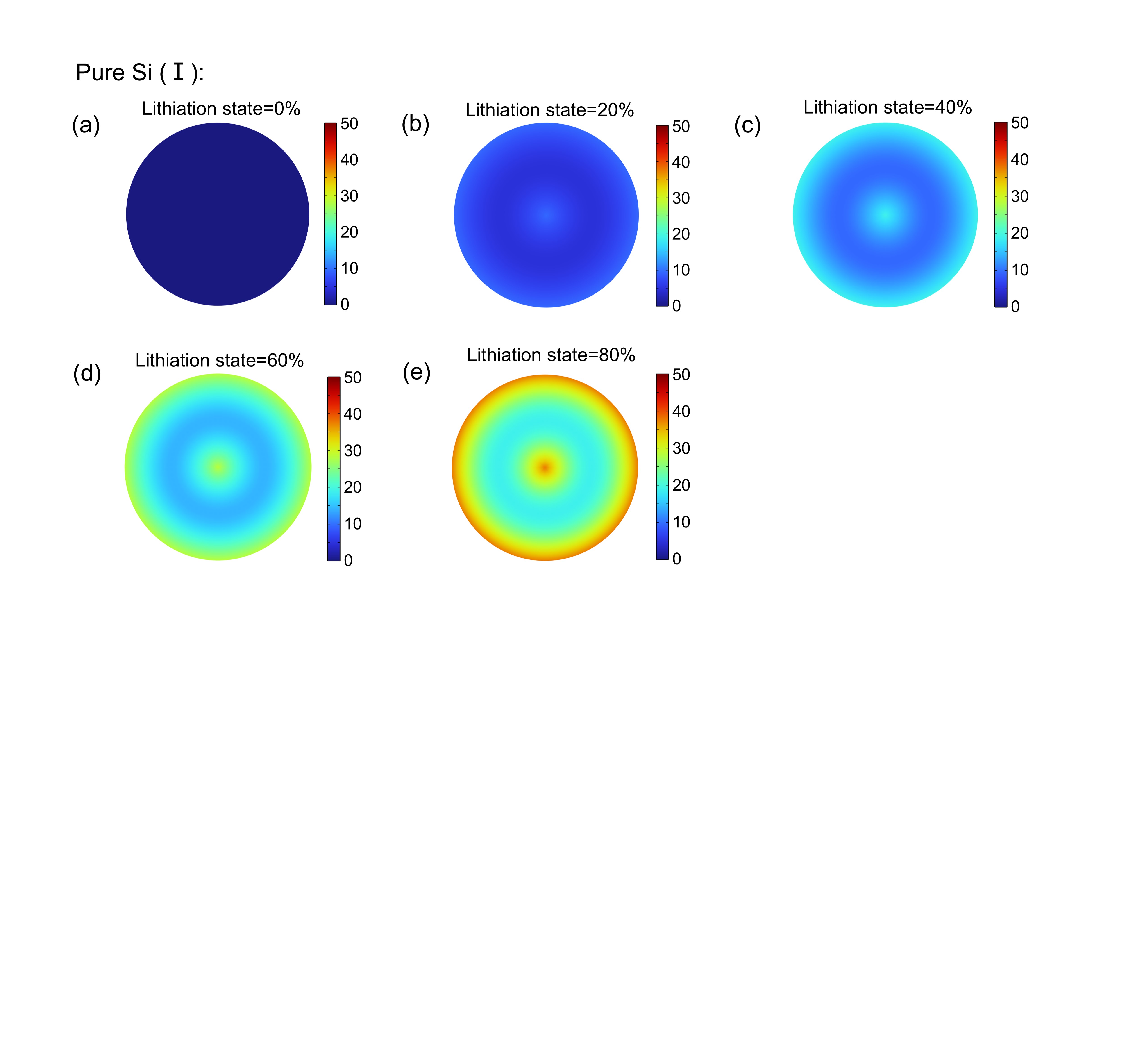


**Fig. S1** Chemo-mechanical model of stress distribution for pure Si (Ⅰ) within various lithiation state


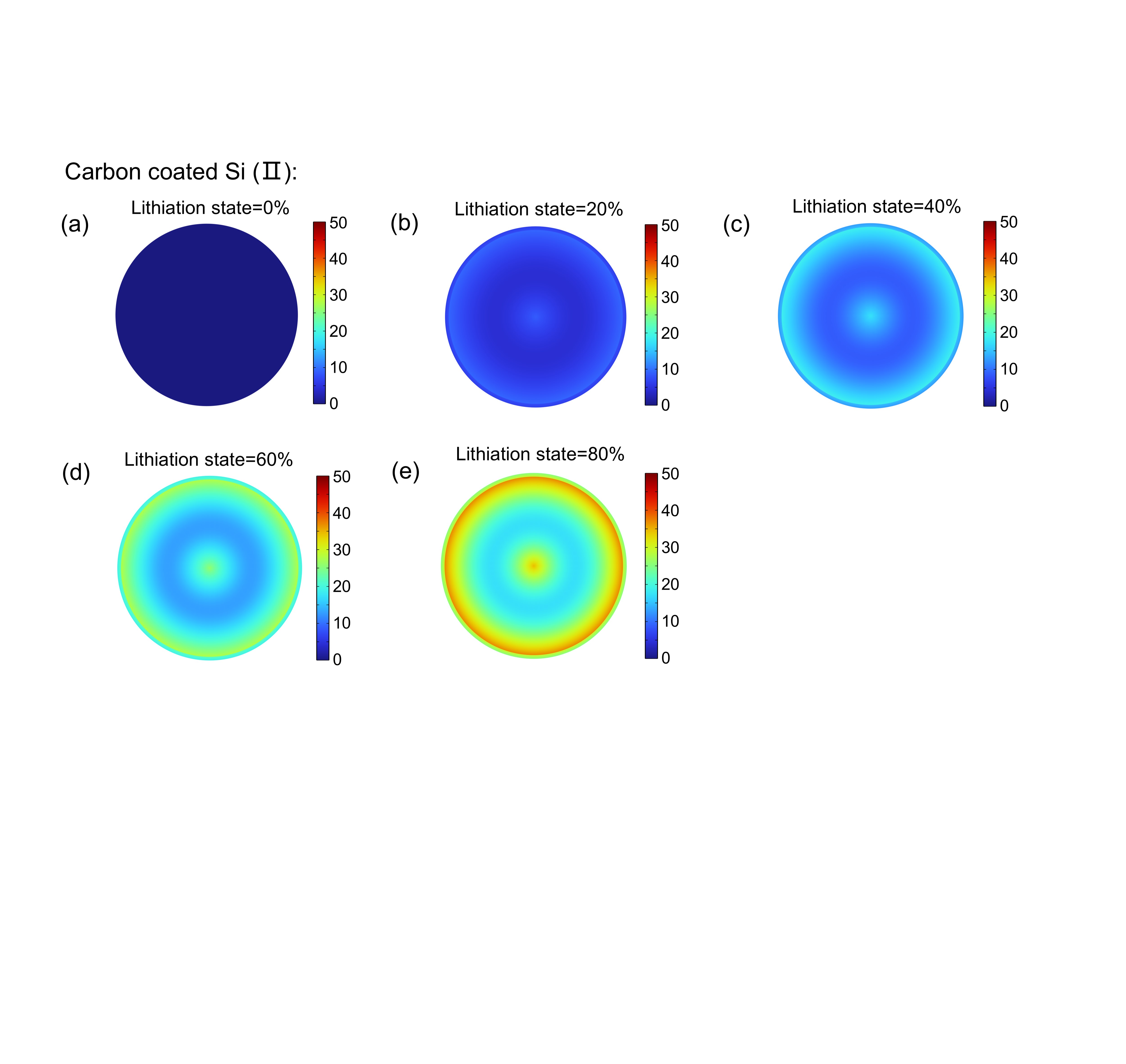


**Fig. S2** Chemo-mechanical model of stress distribution for carbon-coated Si (Ⅱ) within various lithiation state


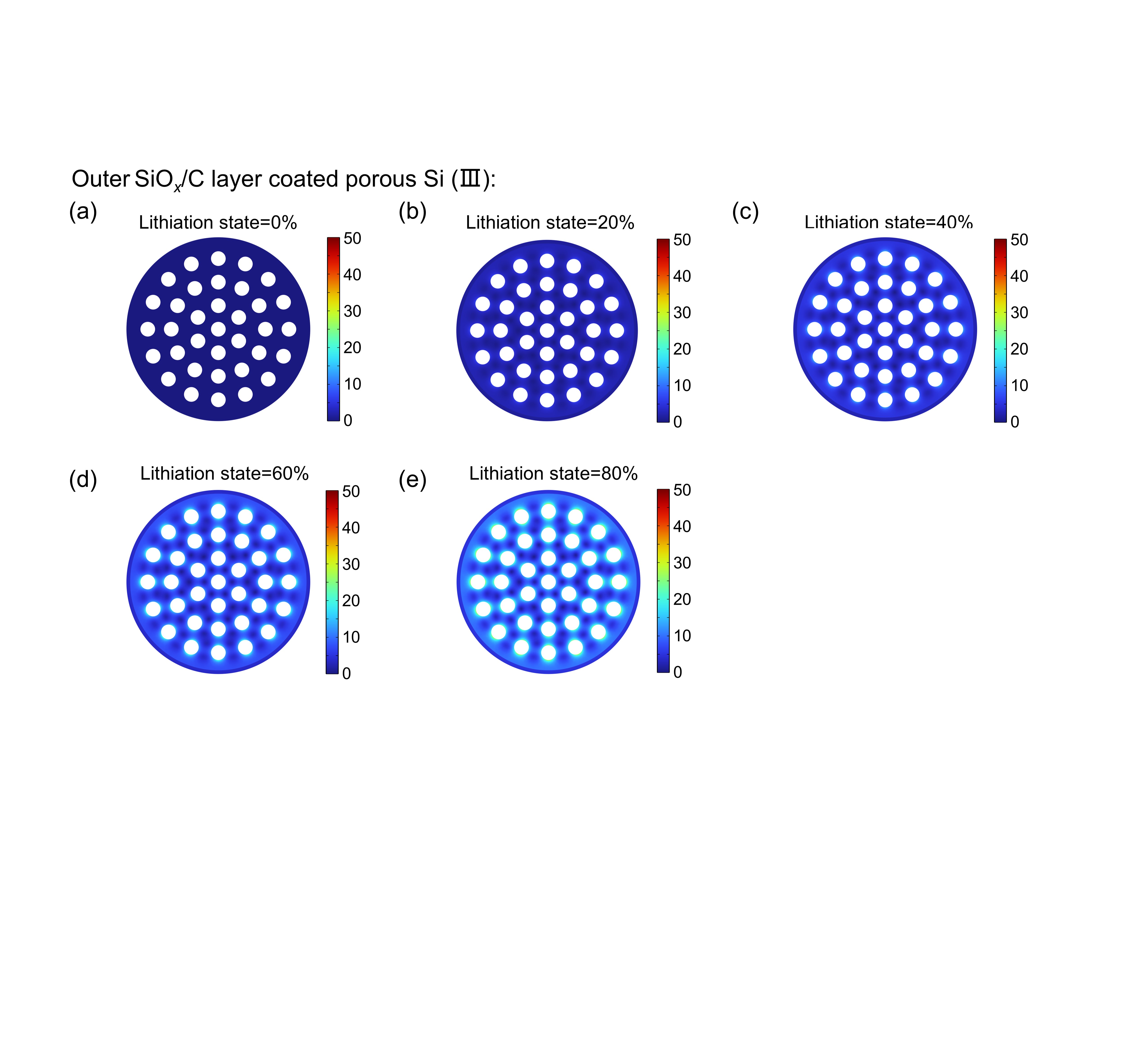


**Fig. S3** Chemo-mechanical model of stress distribution for outer SiO*_x_*/C layer coated porous Si (Ⅲ) within various lithiation state


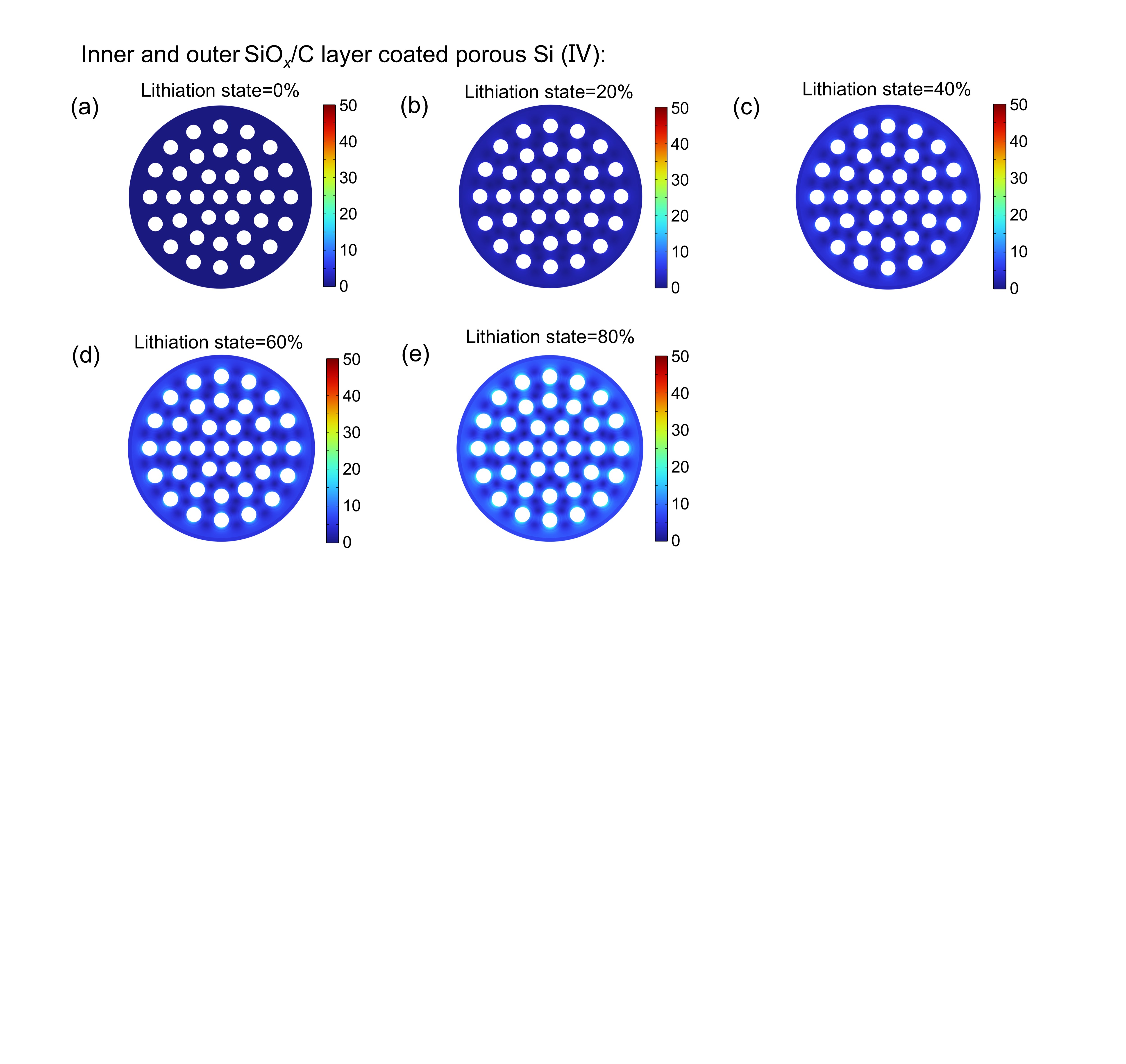


**Fig. S4** Chemo-mechanical model of stress distribution for both inner and outer SiO*_x_*/C layer coated porous Si (Ⅳ) within various lithiation state


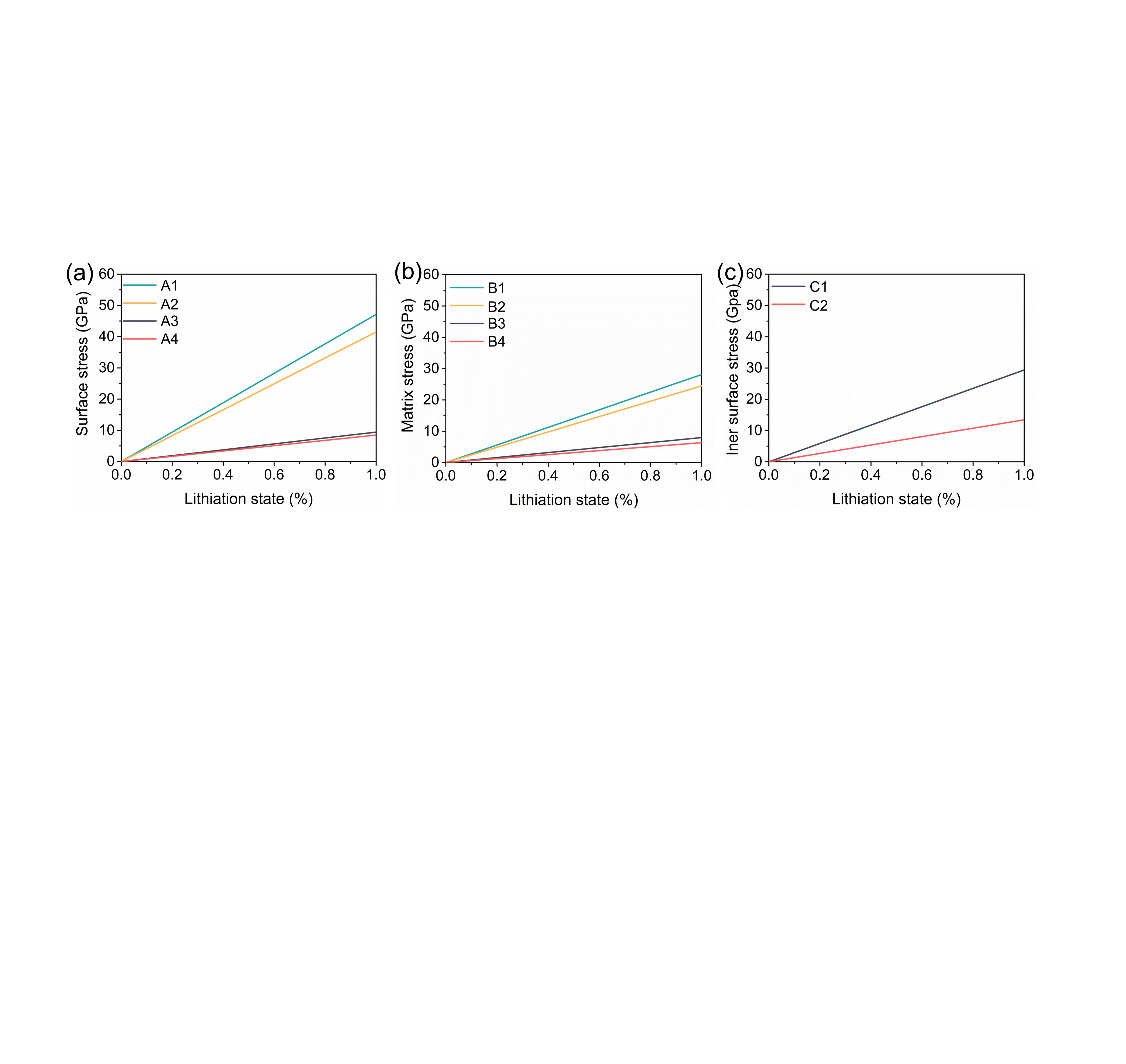


**Fig. S5** (**a**) Surface stress and (**b**) Si matrix stress distribution for various materials upon lithiation. (**c**) Inner pore surface stress distribution of outer SiO*_x_*/C layer coated porous Si (Ⅲ, C1) and both inner and outer SiO*_x_*/C layer coated porous Si (Ⅳ, C2) upon lithiation


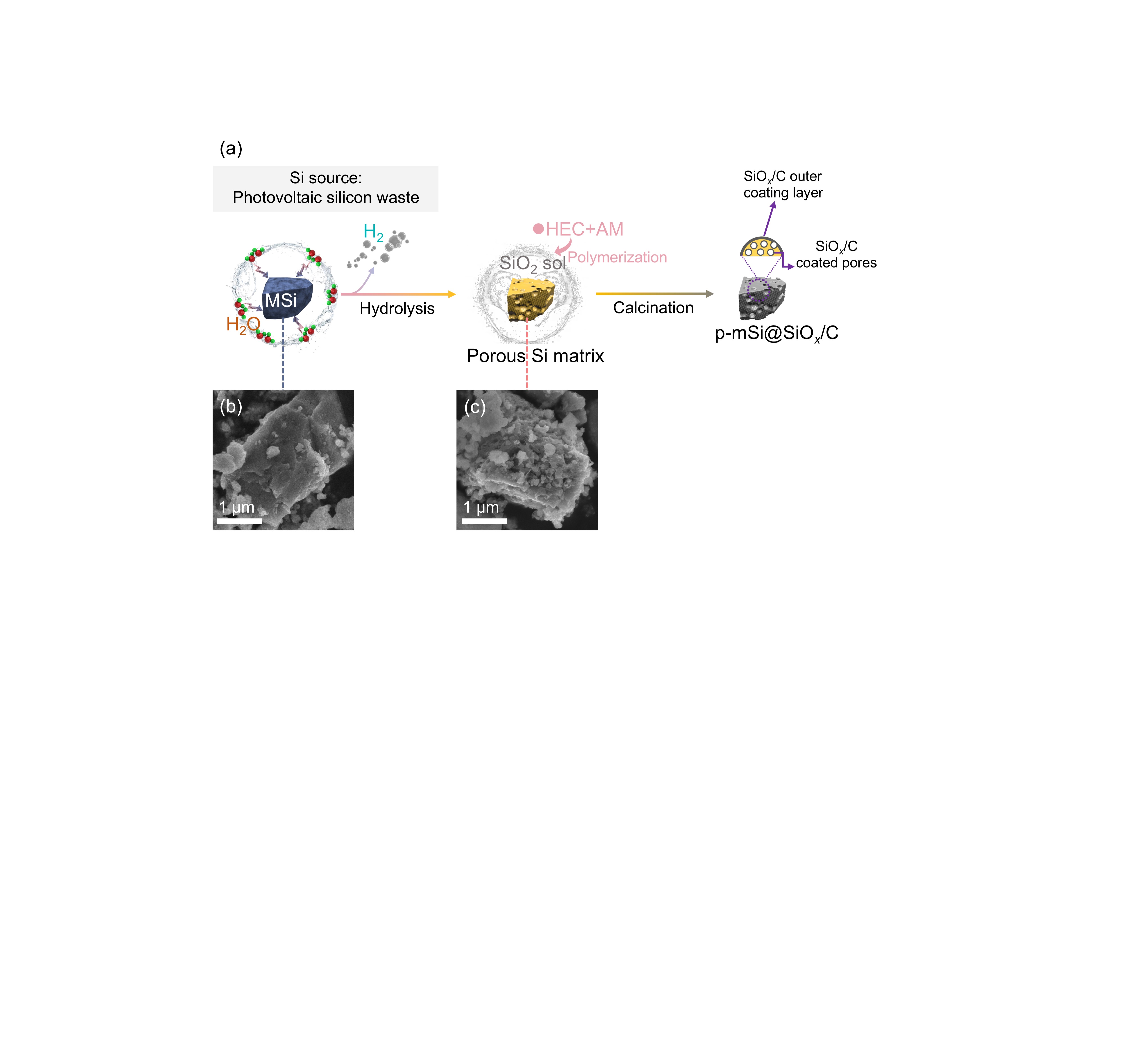


**Fig. S6** (**a**) Schematic illustration of the preparation process for the p-mSi@SiO*_x_*/C. SEM images of (**b**) MSi and (**c**) the obtained porous Si matrix from the hydrolysis of MSi within 1 h


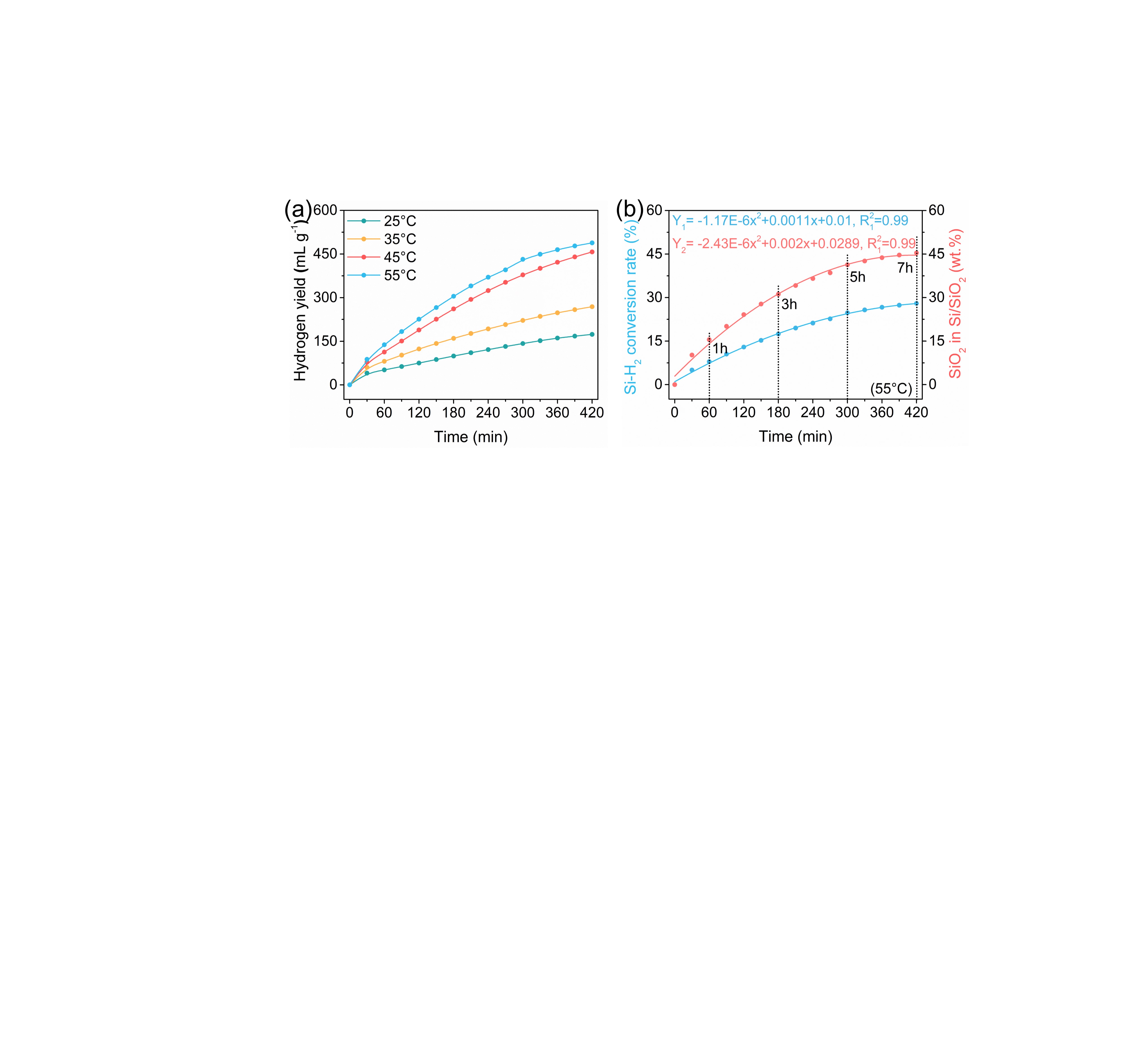


**Fig. S7** (**a**) Hydrogen evolution performance of MSi at different temperatures. (**b**) The Si-H_2_ conversion rate curve of MSi, and the corresponding SiO_2_ content in Si/SiO_2_ mixture

**Note S1:**

As shown in **Fig.** S7a, the hydrogen evolution performance of MSi is positively correlated with reaction temperature. In our specific case of 55℃, it not only can enhance the hydrogen evolution kinetics, but also be compatible for facilitating the subsequent hybrid polymerization process between the hydrolysis-obtained SiO_2_ sol and the organics of HEC and AM. Moreover, based on the hydrolysis equation of $\text{Si+}\text{2H}_{\text{2}}\text{O →Si}\text{O}_{\text{2}}\text{+2}\text{H}_{\text{2}}$, the Si-H_2_ conversion rate and corresponding SiO_2_ content in the mixture can be calculated and fitted against the reaction time (**Fig.** S7b), indicating the SiO_2_ content in the subsequent SiO*_x_*/C layer can be well-controlled through adjusting the hydrolysis reaction, further balancing the electrochemical capacity and cyclic stability.


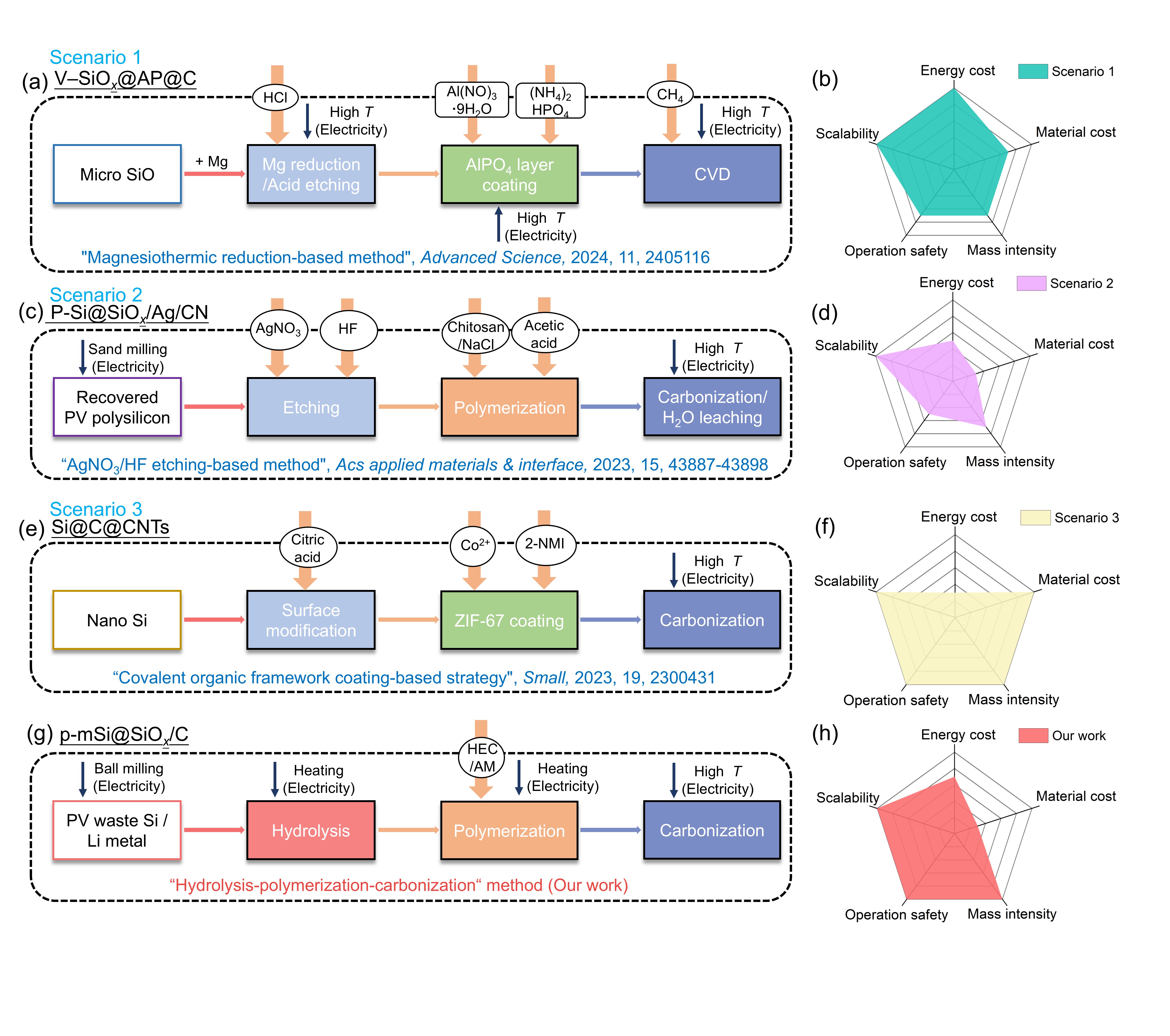


**Fig. S8** Flow diagrams and comprehensive analysis of four scenarios for advanced Si anode preparation, namely, (**a, b**) the magnesiothemic reduction-based method (scenario 1), (**c, d**) the AgNO_3_/HF etching-based method (scenario 2), (**e, f**) covalent organic framework coating strategy (scenario 3) and (**g, h**) our developed “hydrolysis-polymerization-carbonization” method


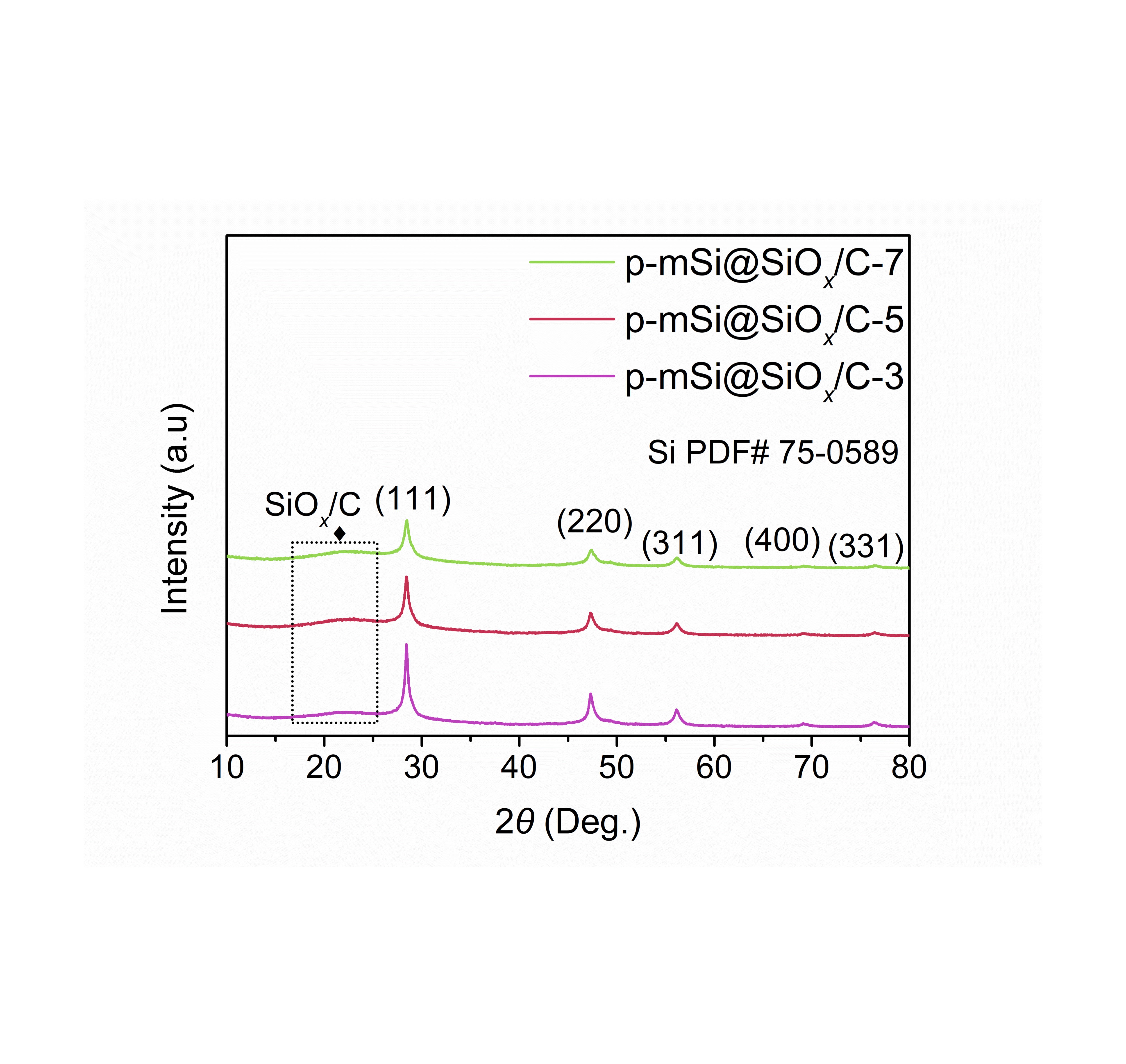


**Fig. S9** XRD patterns of p-mSi@SiO*_x_/*C-3, p-mSi@SiO*_x_*/C-5, p-mSi@SiO*_x_*/C-7


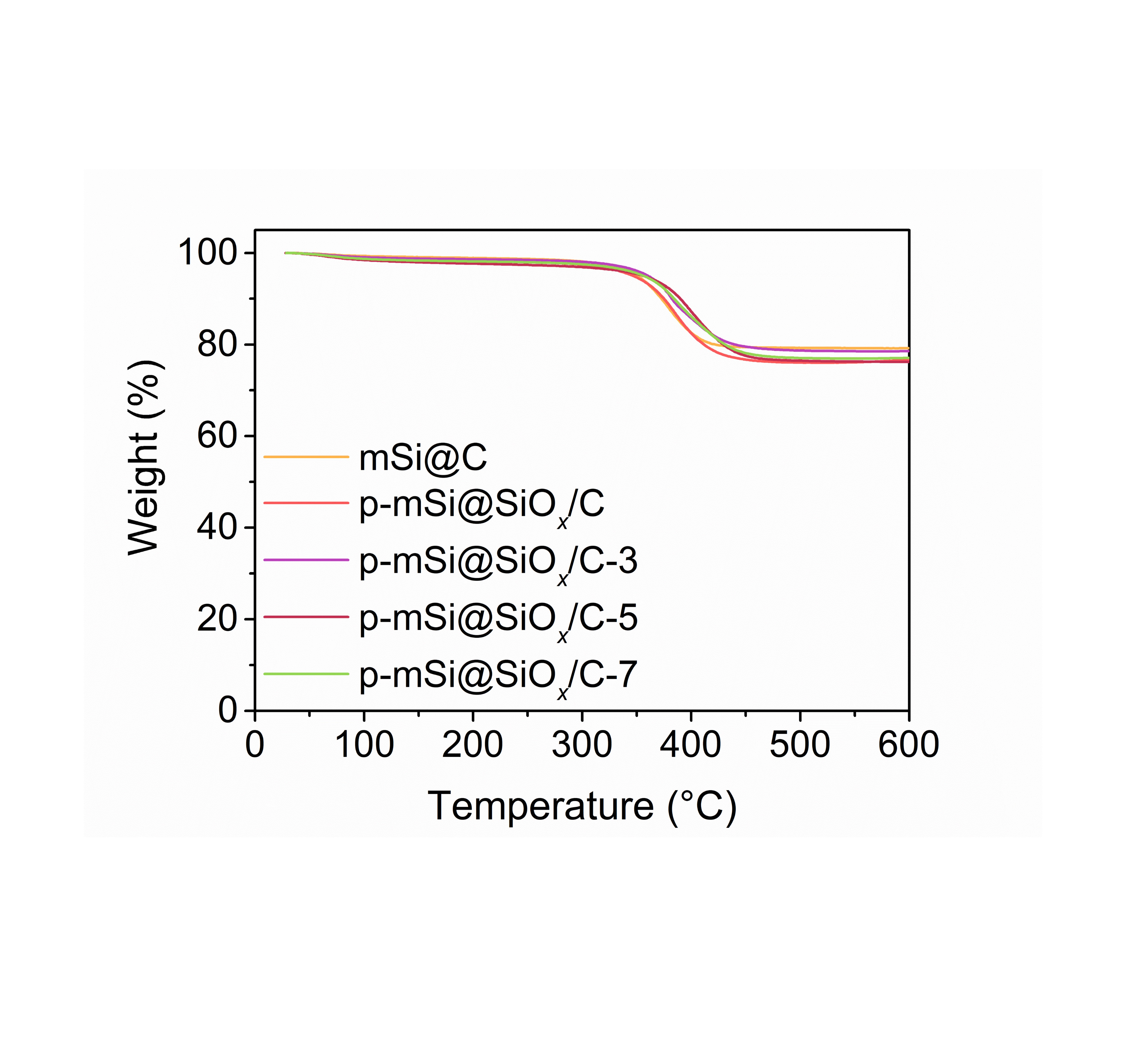


**Fig. S10** TG curves of mSi@C, p-mSi@SiO*_x_*/C, p-mSi@SiO*_x_*/C-3, p-mSi@SiO*_x_*/C-5, p-mSi@SiO*_x_*/C-7


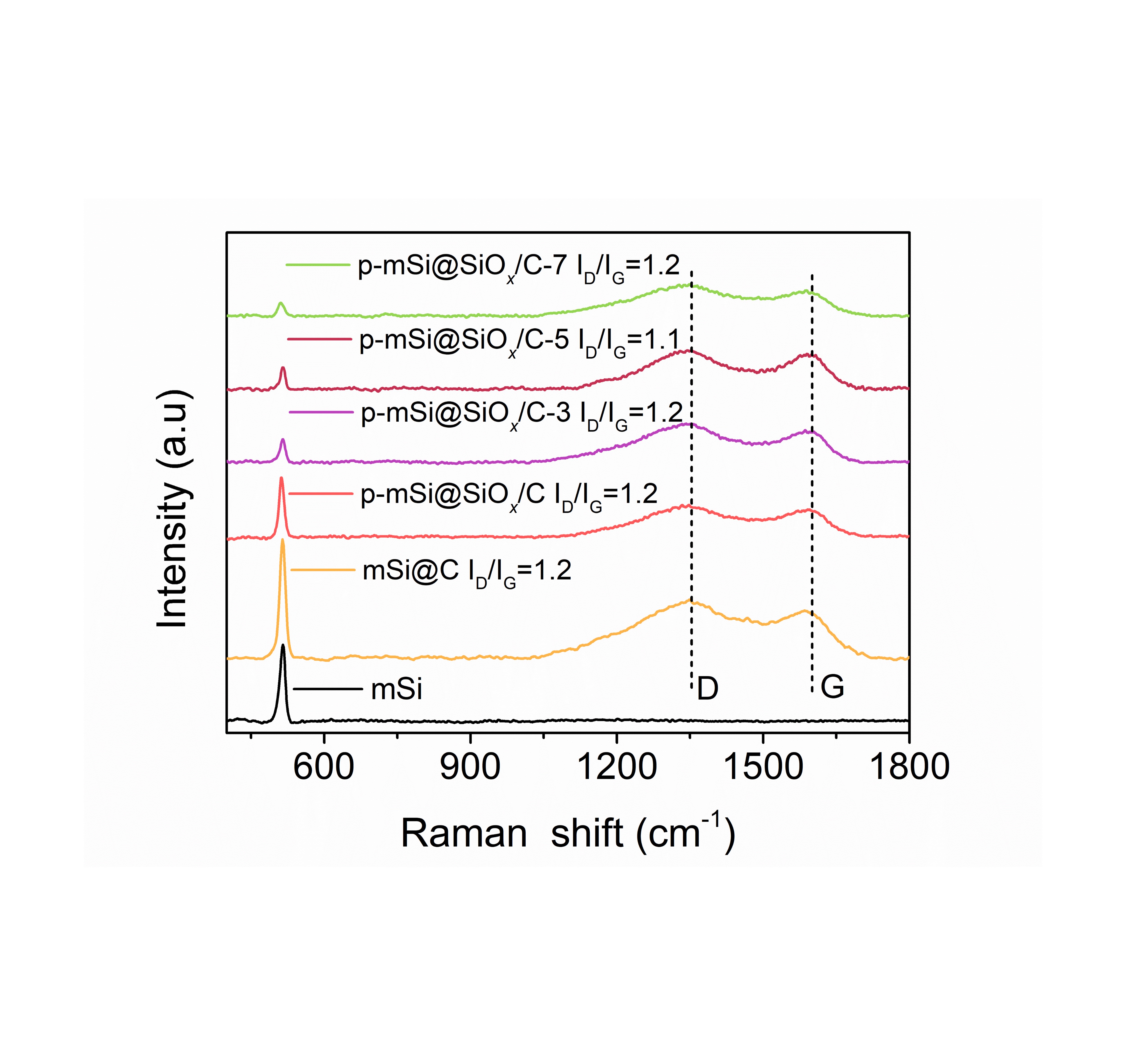


**Fig. S11** Raman spectra of mSi, mSi@C, p-mSi@SiO*_x_*/C, p-mSi@SiO*_x_*/C-3, p-mSi@SiO*_x_*/C-5, p-mSi@SiO*_x_*/C-7


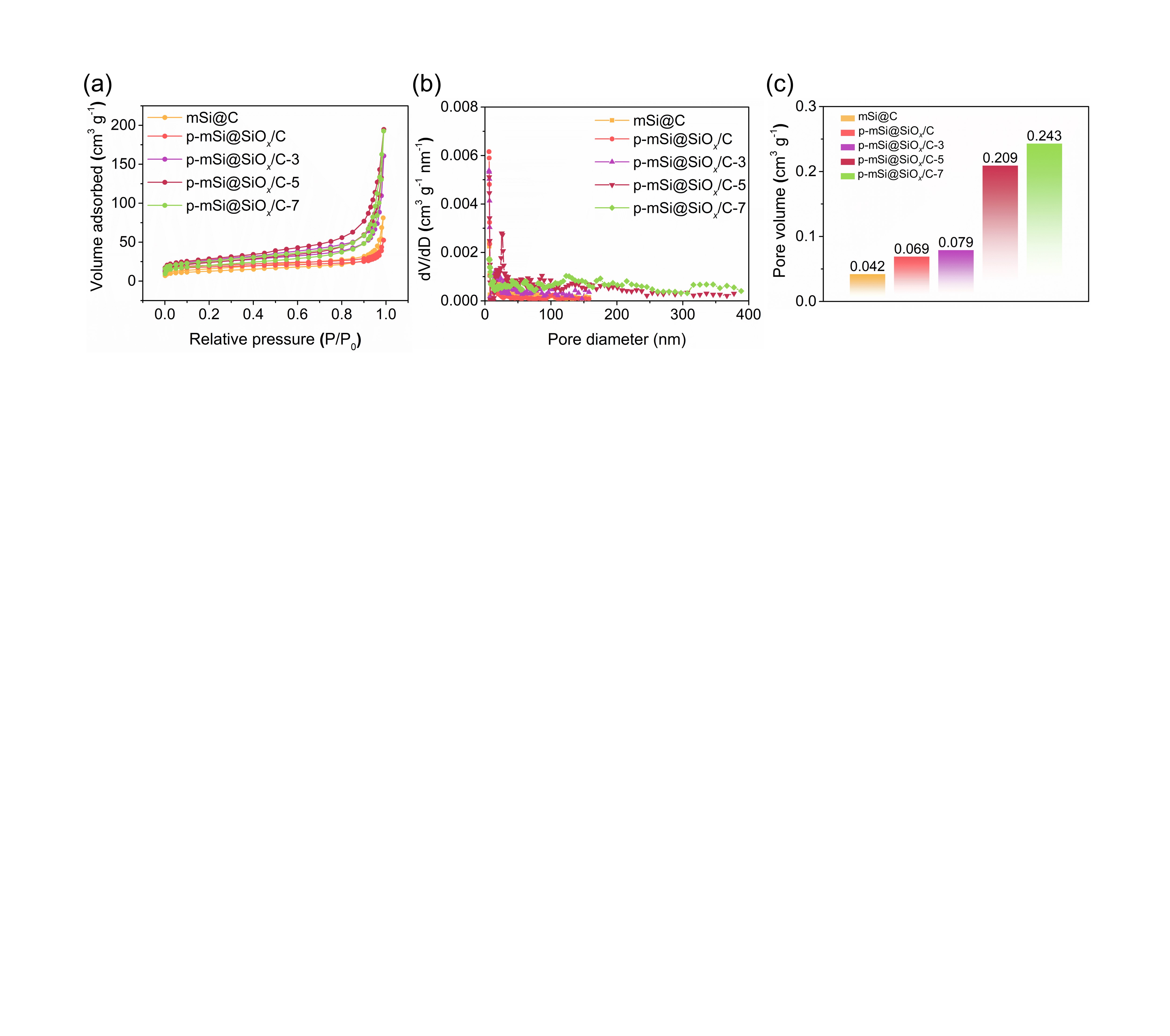


**Fig. S12** (**a**) Nitrogen sorption isotherms curves, (**b**) pore size distribution and (**c**) pore volume of mSi@C, p-mSi@SiO*_x_*/C, p-mSi@SiO*_x_*/C-3, p-mSi@SiO*_x_*/C-5 and p-mSi@SiO*_x_*/C-7


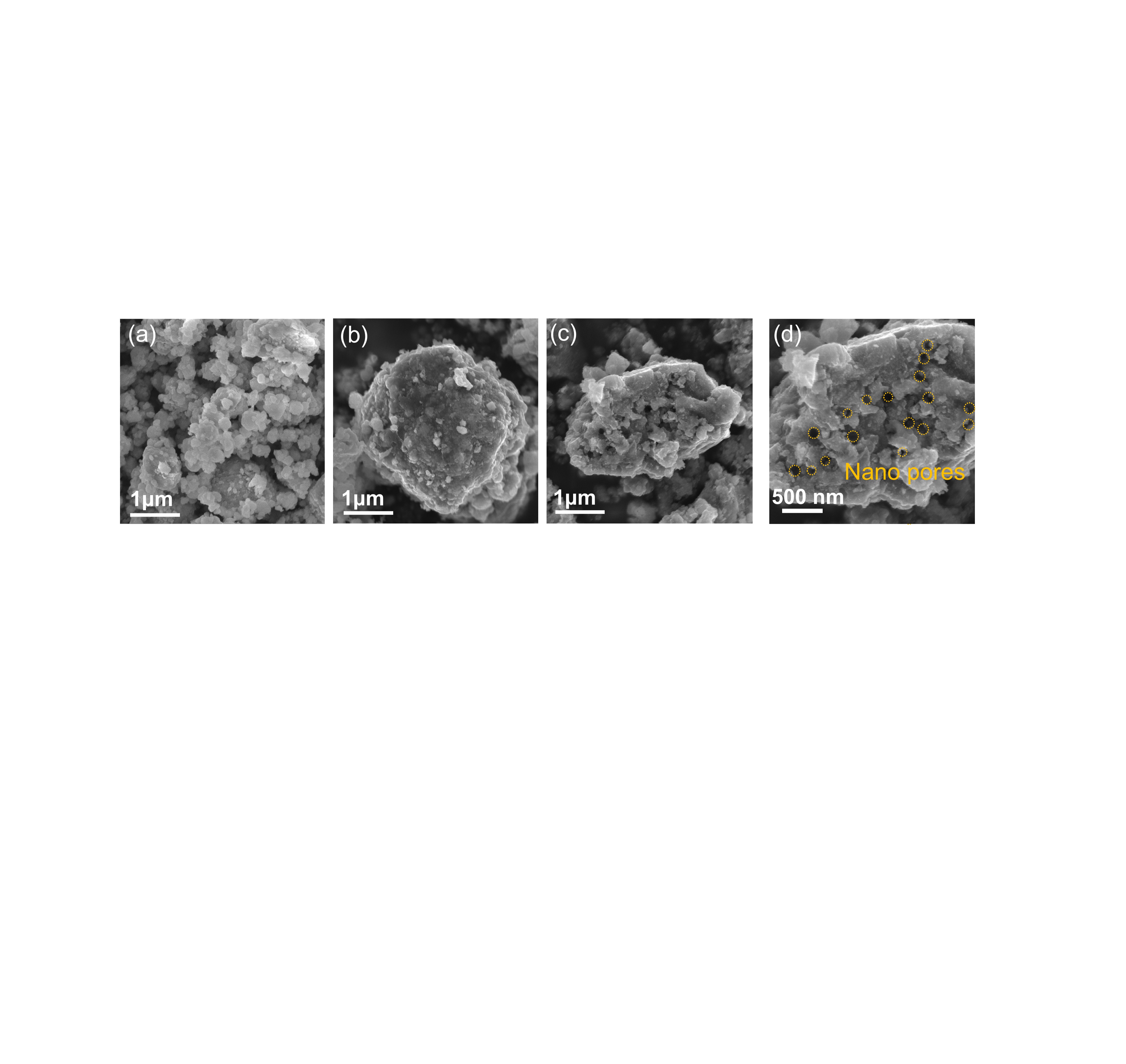


**Fig. S13** SEM images of (**a**) mSi, (**b**) mSi@C and (**c, d**) p-mSi@SiO*_x_*/C


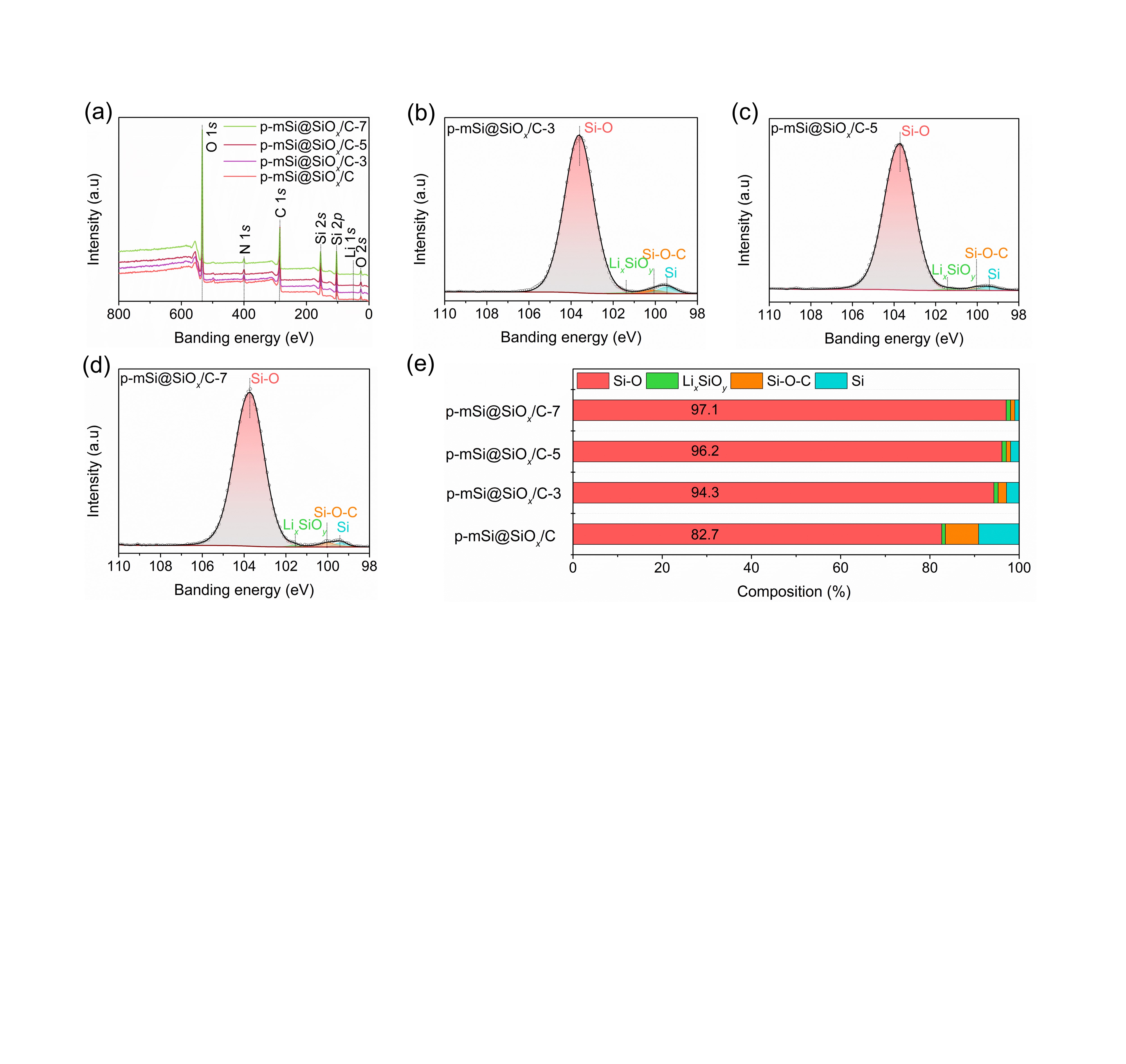


**Fig. S14** (**a**) XPS survey spectra of p-mSi@SiO*_x_*/C, p-mSi@SiO*_x_*/C-3, p-mSi@SiO*_x_*/C-5, p-mSi@SiO*_x_*/C-7. High resolution Si 2*p* spectra of (**b**) p-mSi@SiO*_x_*/C-3, (**c**) p-mSi@SiO*_x_*/C-5 and (**d**) p-mSi@SiO*_x_*/C-7. (**e**) The surface composition analysis of Si 2*p* spectra for p-mSi@SiO*_x_*/C, p-mSi@SiO*_x_*/C-3, p-mSi@SiO*_x_*/C-5, p-mSi@SiO*_x_*/C-7


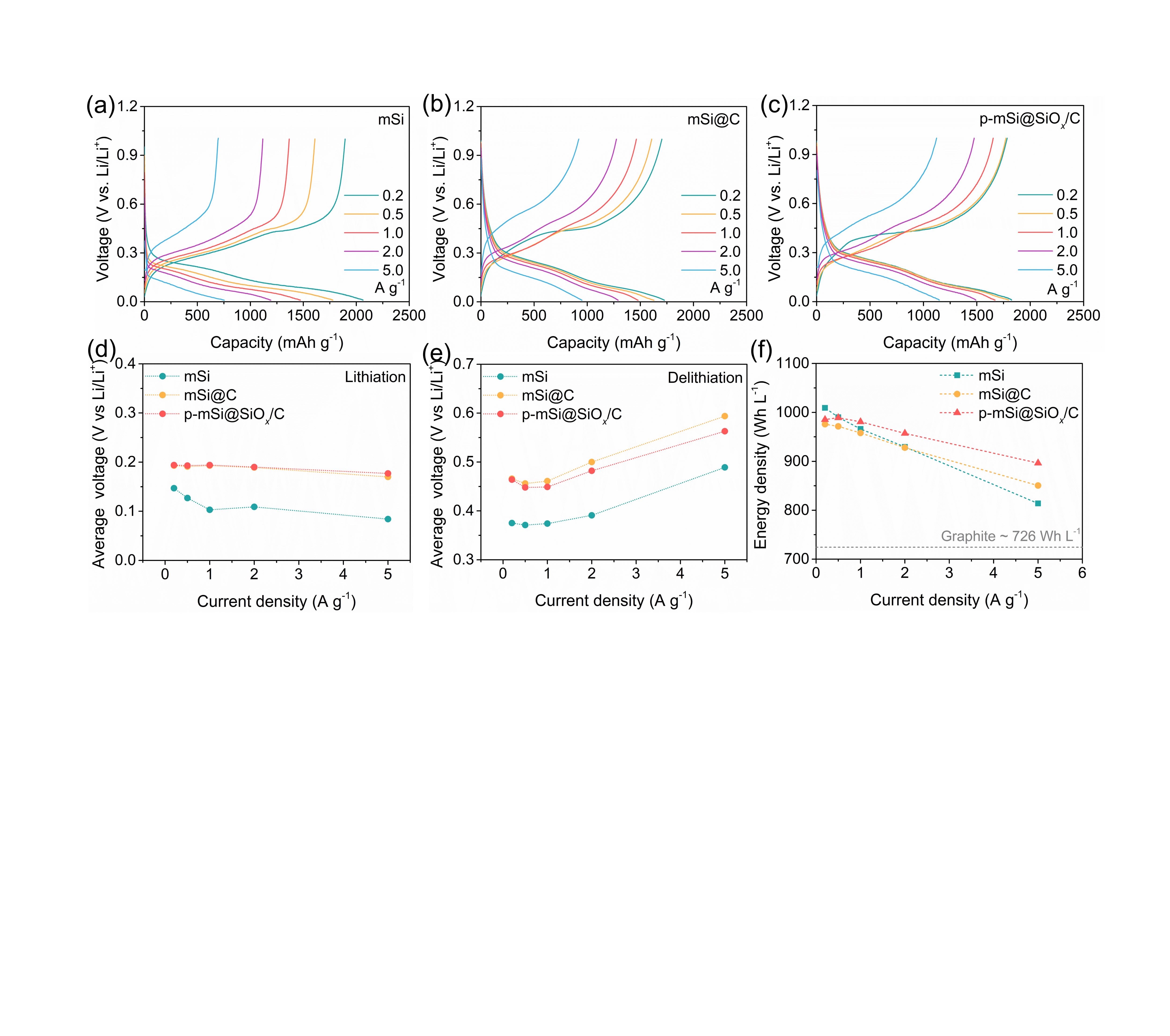


**Fig. S15** Discharge-charge profiles of (**a**) mSi, (b) mSi@C and (**c**) p-mSi@SiO*_x_*/C at various current densities. (**d, e**) Average lithiation/delithiation voltages and (**f**) energy densities of mSi, mSi@C and p-mSi@SiO*_x_*/C at various current densities


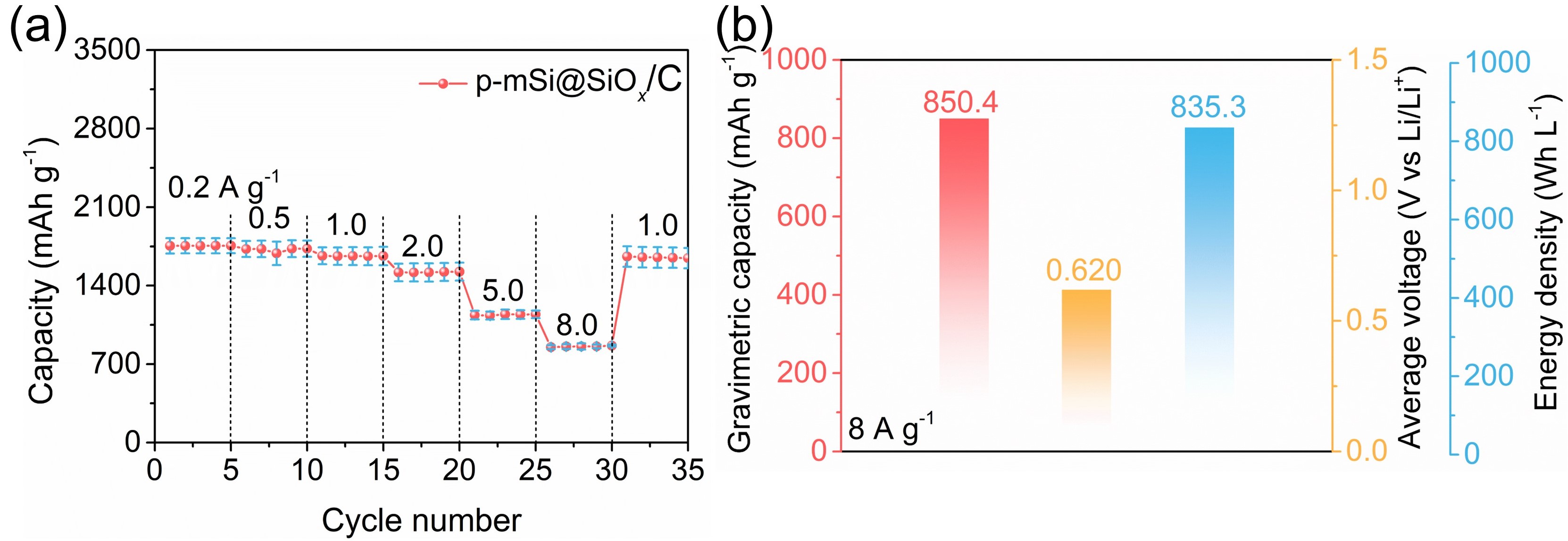


**Fig. S16** (**a**) Rate performance of p-mSi@SiO*_x_*/C at 0.2-8 A g⁻^1^. (**b**) Reversible gravimetric capacity, average lithiation voltage and stack cell energy density of p-mSi@SiO*_x_*/C at 8 A g⁻^1^


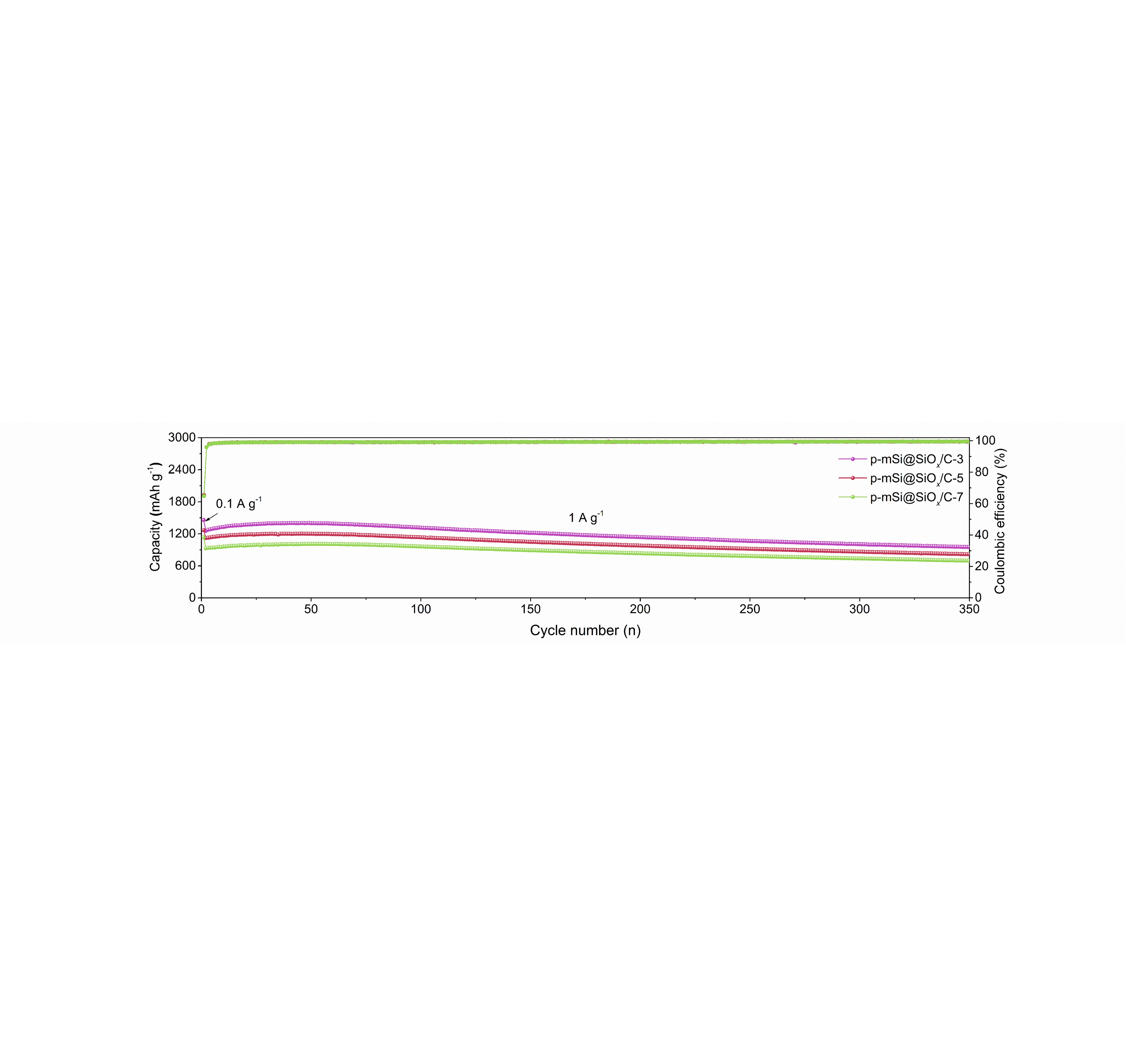


**Fig. S17** Cyclic performance of p-mSi@SiO*_x_*/C-3, p-mSi@SiO*_x_*/C-5 and p-mSi@SiO*_x_*/C-7 at 1 A g⁻^1^.

**Note S2:**

As shown in **Fig.** S17, the reversible capacities and retention rates of the p-mSi@SiO*_x_*/C-3, p-mSi@SiO*_x_*/C-5 and p-mSi@SiO*_x_*/C-7 after 350 cycles at 1A g⁻^1^ are 1070.2 mAh g⁻^1^/63.0%, 951.5 mAh g⁻^1^/67.9%, 818.1 mAh g⁻^1^/68.1% and 696.3 mAh g⁻^1^/68.9%, respectively.


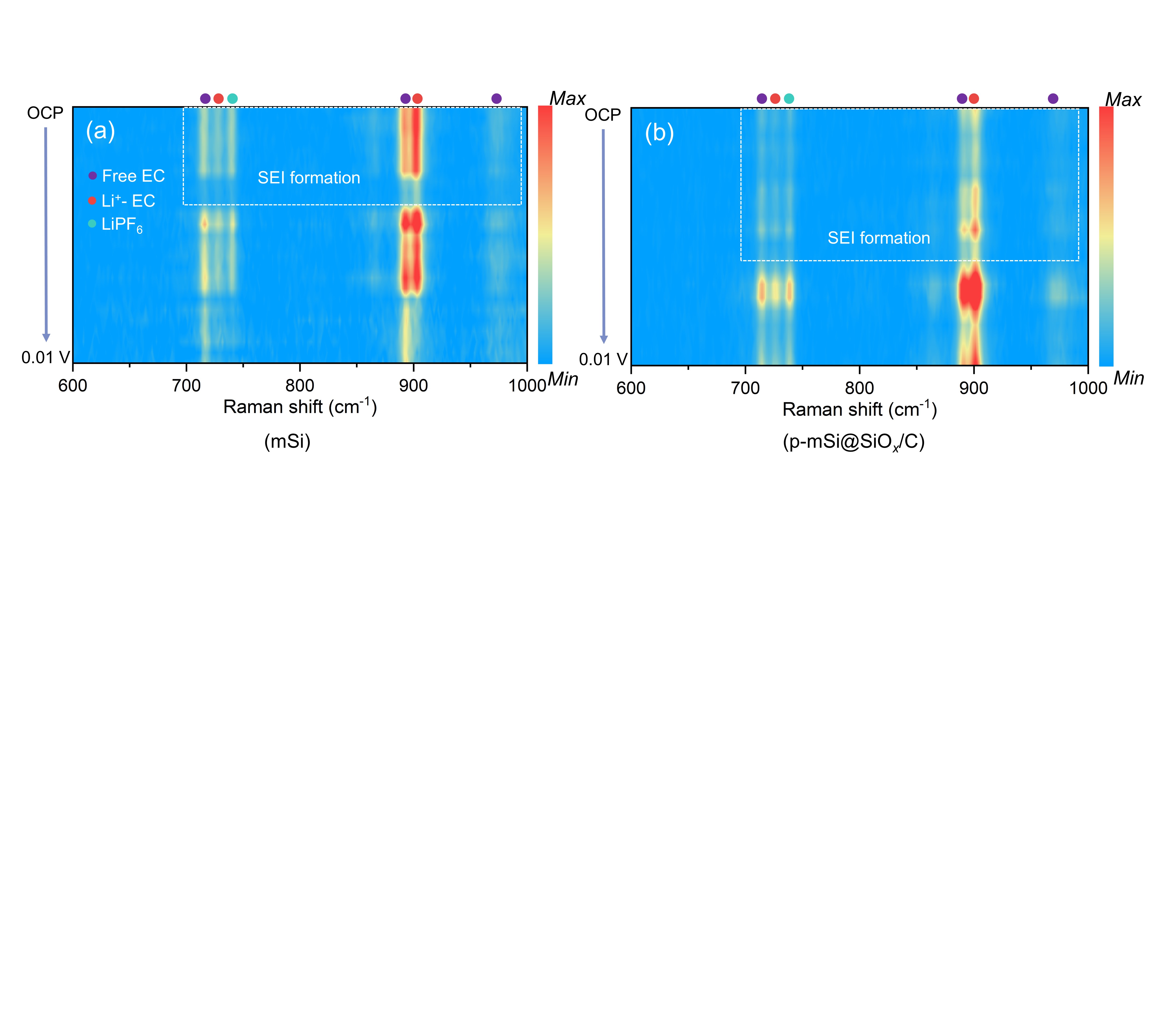


**Fig. S18** In situ Raman tests of (**a**) mSi and (**b**) p-mSi@SiO*_x_*/C electrodes during the first lithiation process


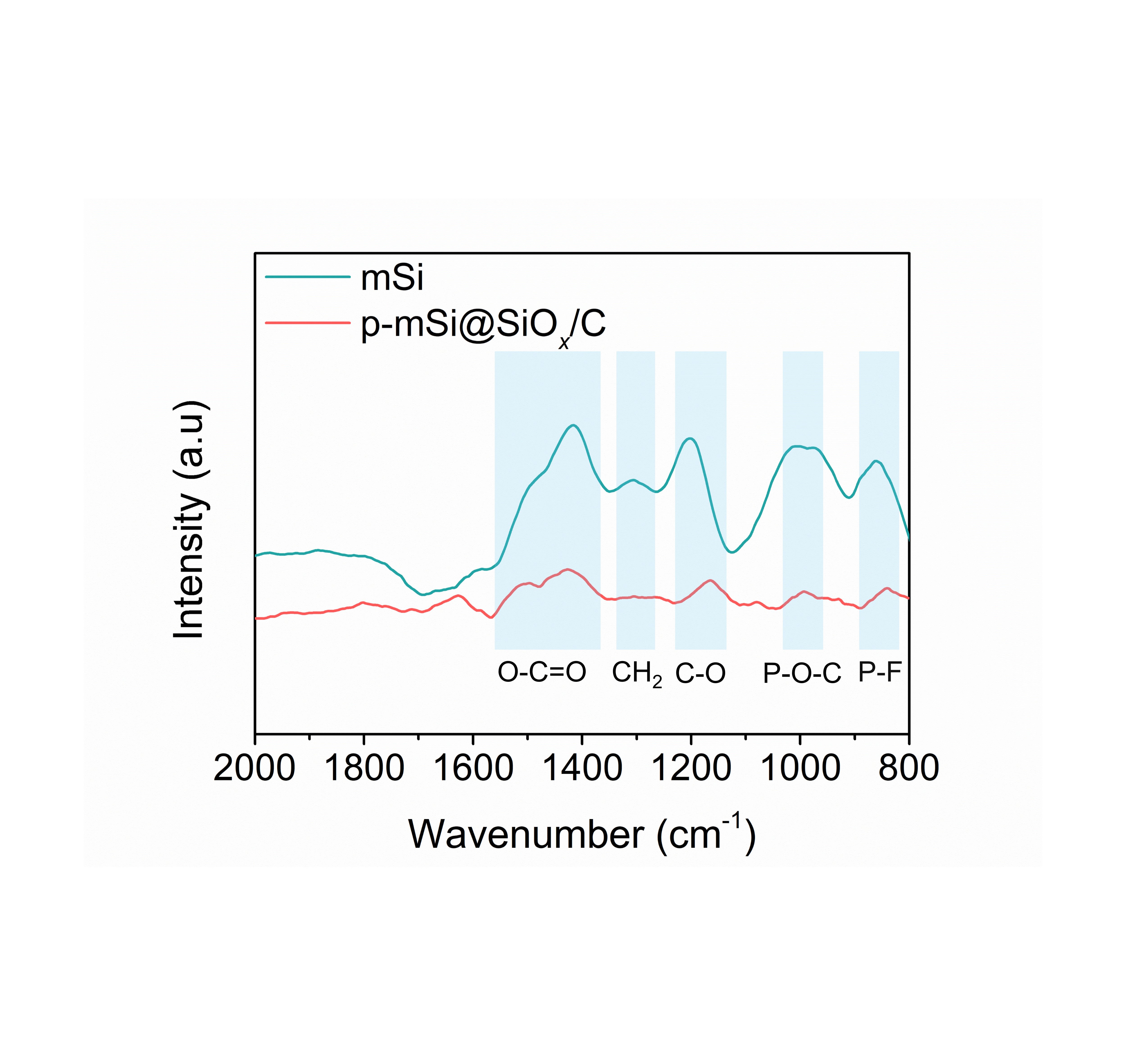


**Fig. S19** FTIR spectra of lithiated mSi and p-mSi@SiO*_x_*/C electrodes


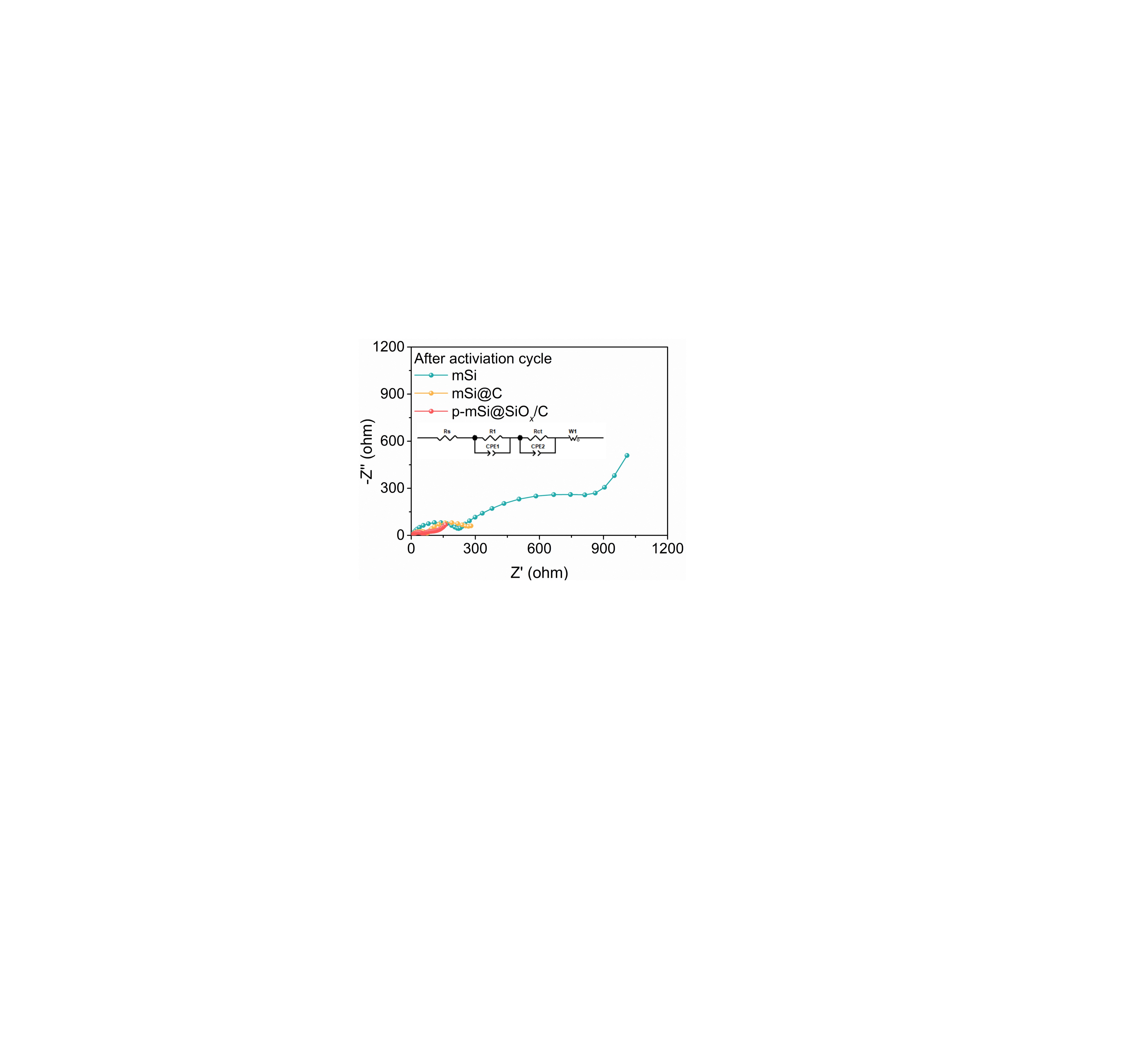


**Fig. S20** Nyquist plots of mSi, mSi@C and p-mSi@SiO*_x_*/C after the activation cycle. The inset is Fitting model of Nyquist plots


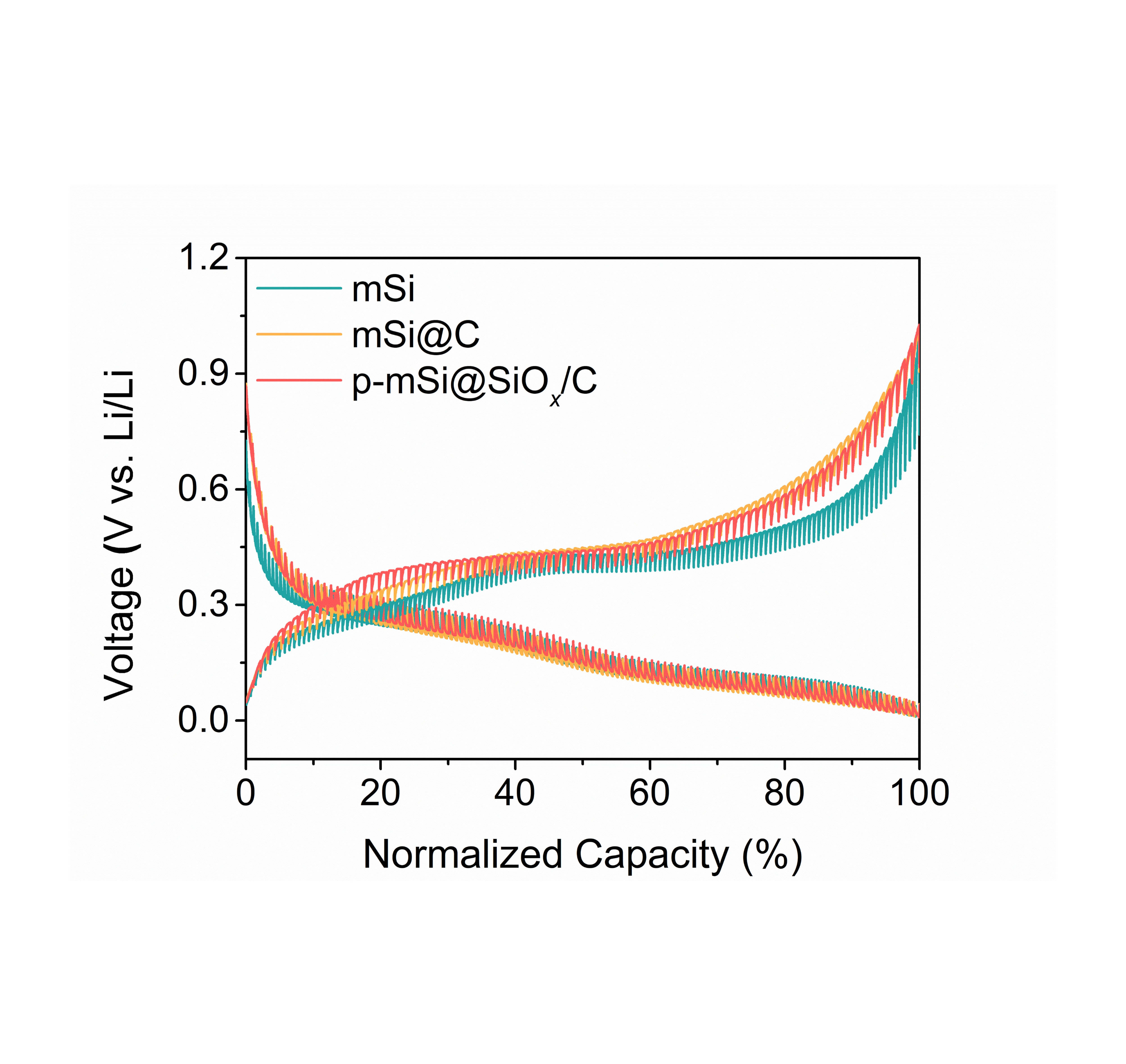


**Fig. S21** GITT curves of lithiation and delithiation process for the mSi, mSi@C and p-mSi@SiO*_x_*/C


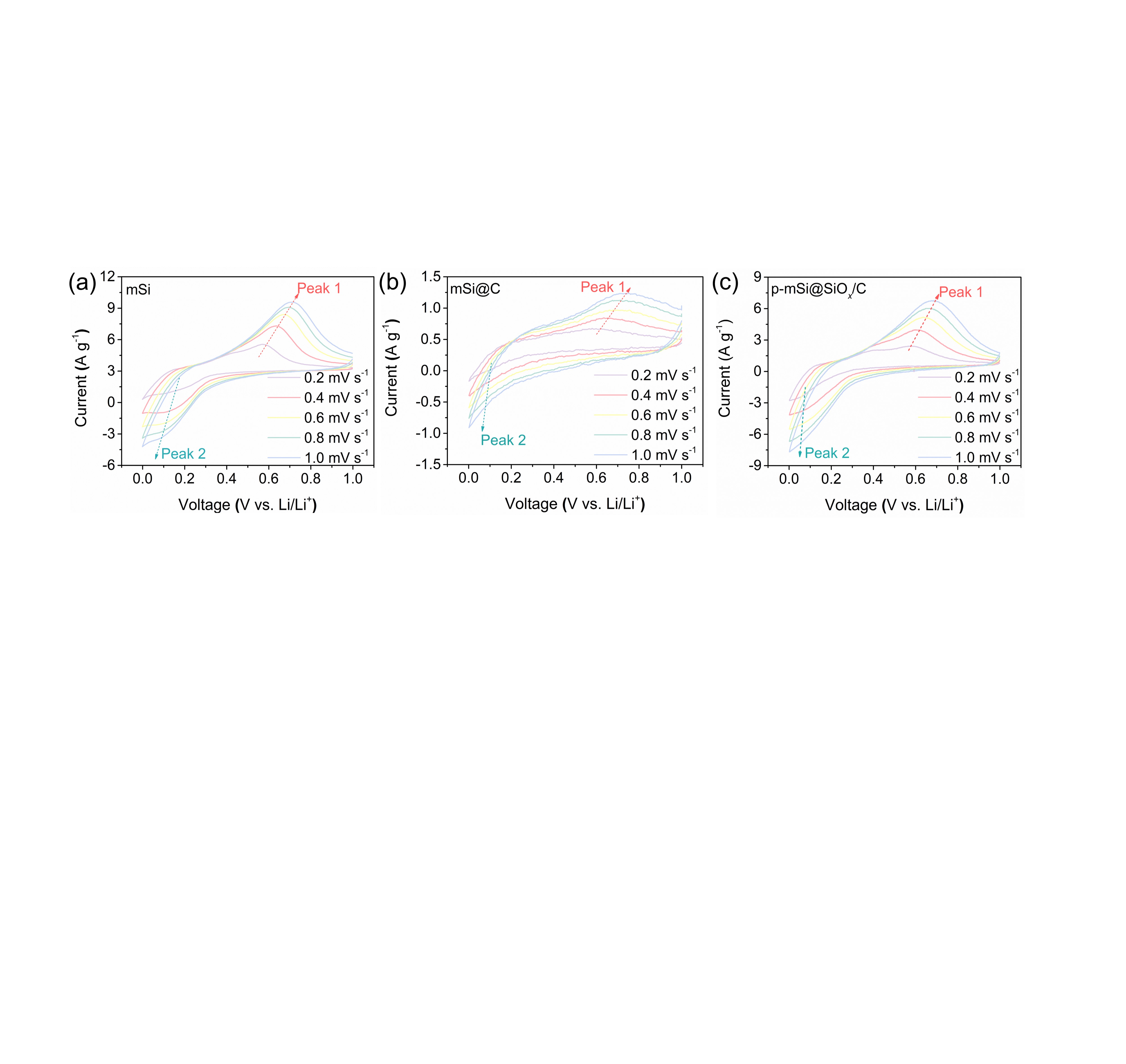


**Fig. S22** CV curves of (**a**) mSi, (**b**) mSi@C and (**c**) p-mSi@SiO*_x_*/C conducted with 0.2~1.0 mV s⁻^1^


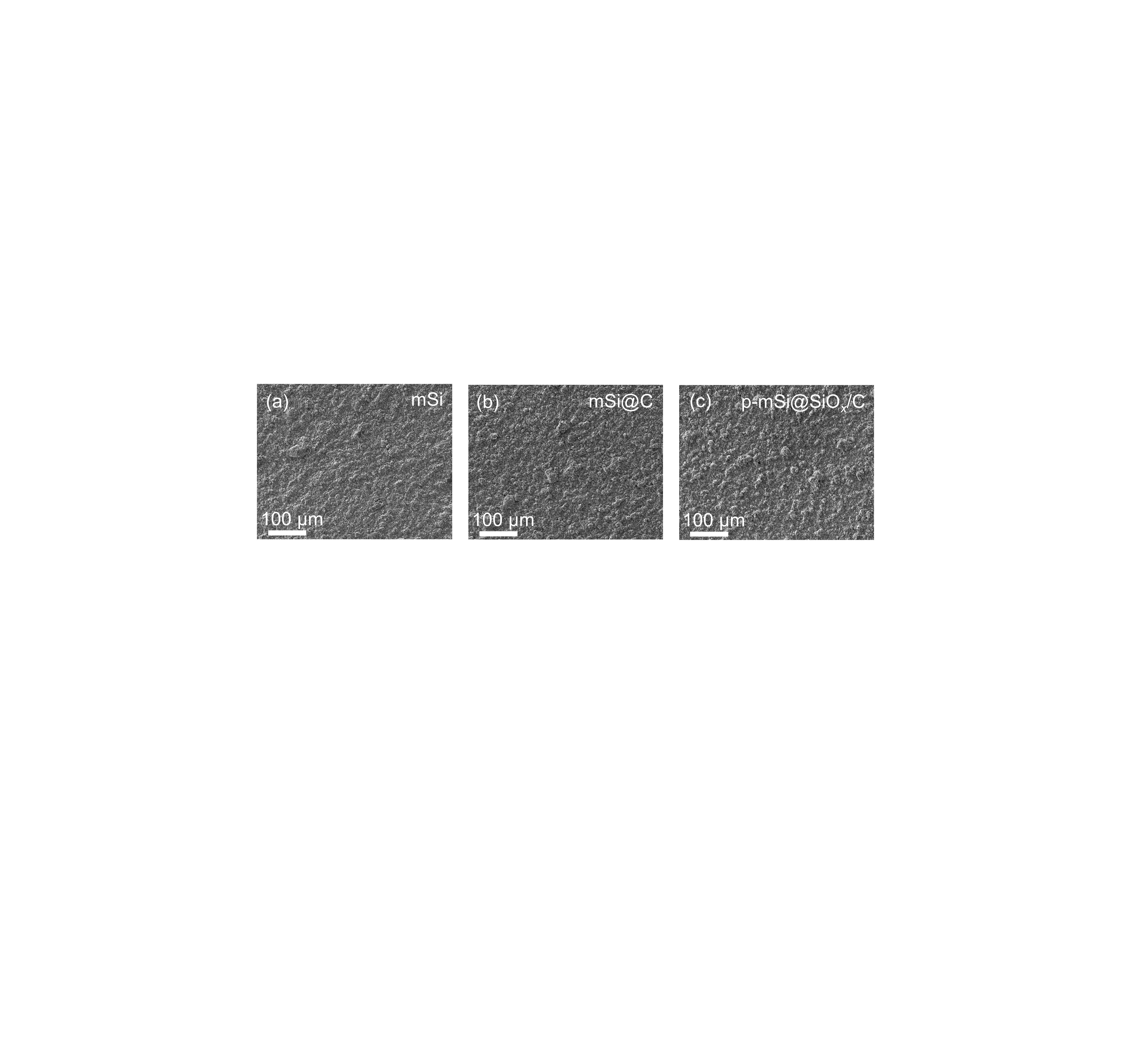


**Fig. S23** Surface SEM images of pristine (**a**) mSi, (**b**) mSi@C and (**c**) p-mSi@SiO*_x_*/C electrodes


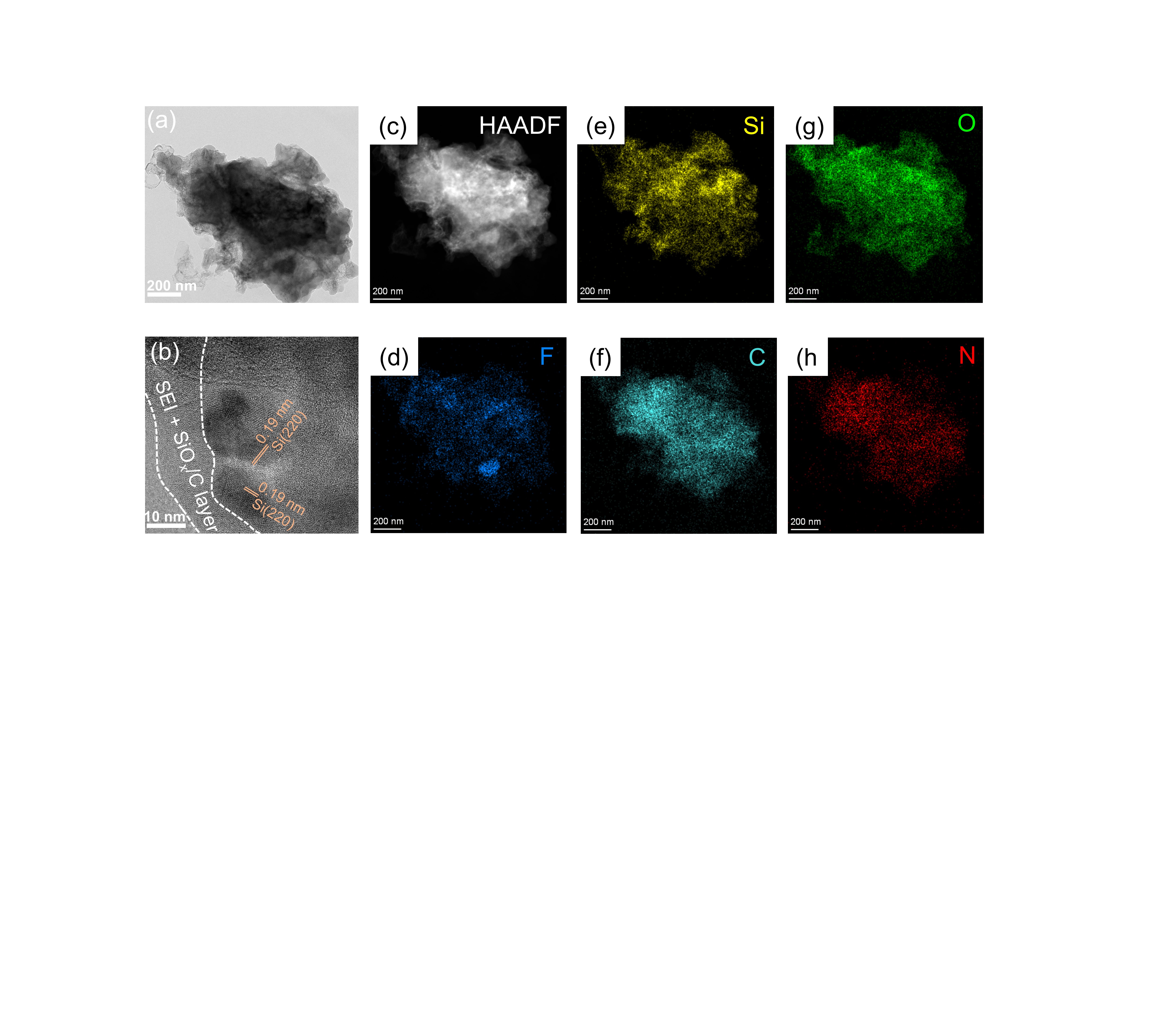


**Fig. S24** (**a, b**) TEM images and (**c-h**) EDS mapping of cycled p-mSi@SiO*_x_*/C


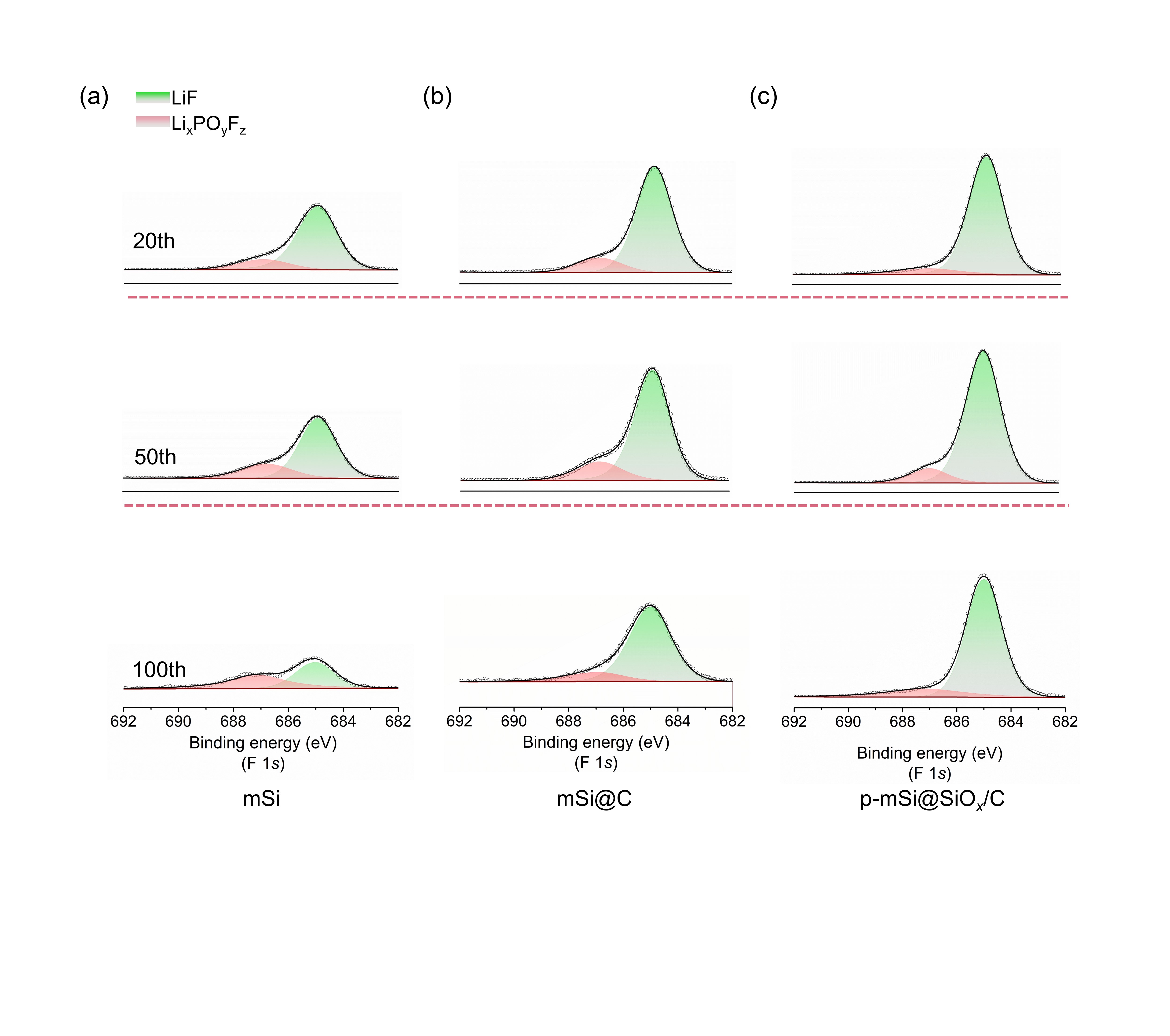


**Fig. S25** High resolution F 1*s* XPS spectra of (**a**) mSi, (**b**) mSi@C and (**c**) p-mSi@SiO*_x_*/C electrodes after 20th, 50th and 100th cycle


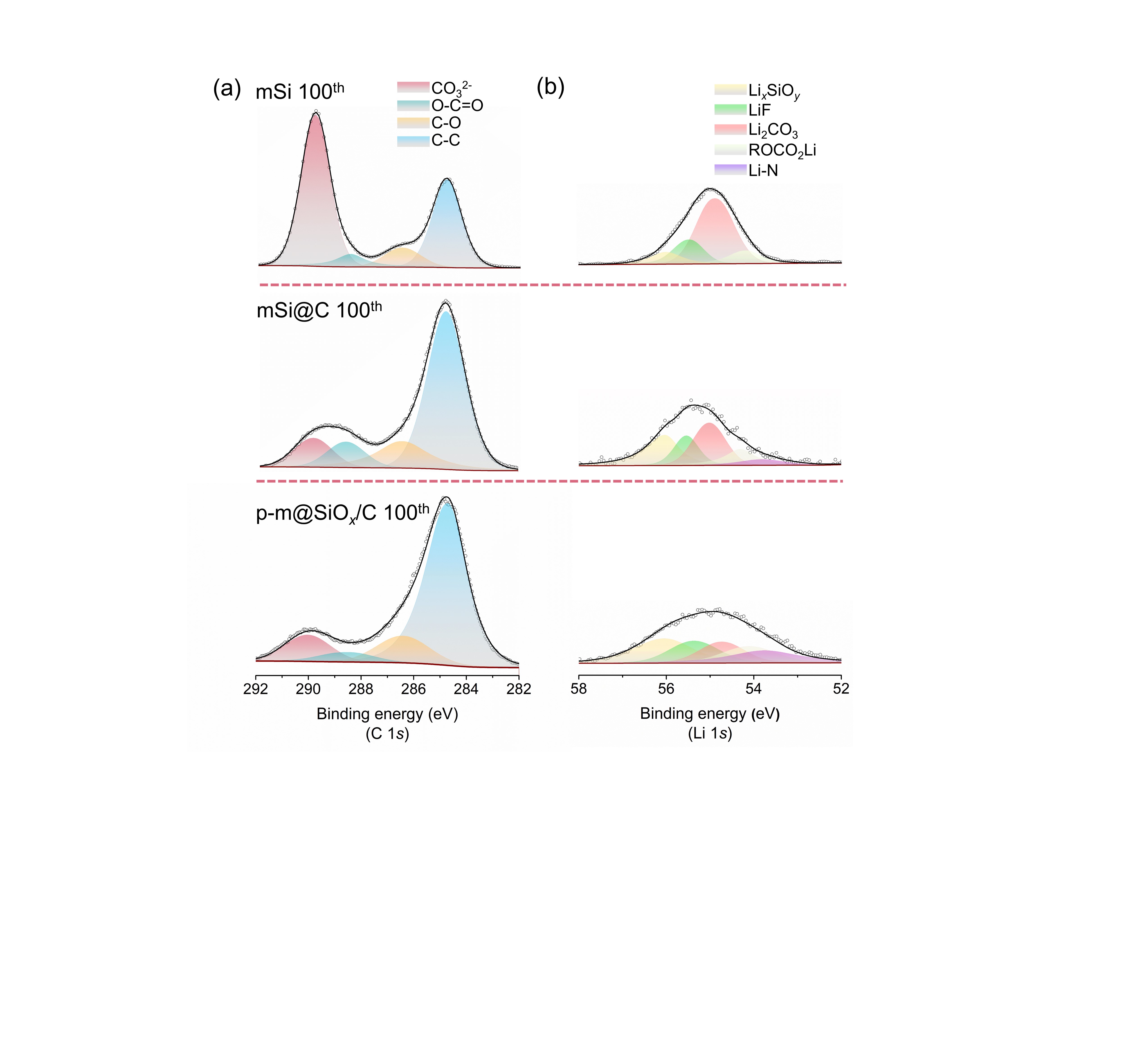


**Fig. S26** High resolution XPS spectra of (**a**) C 1*s*, and (**b**) Li 1*s* for the cycled mSi, mSi@C and p-mSi@SiO*_x_*/C electrodes, respectively


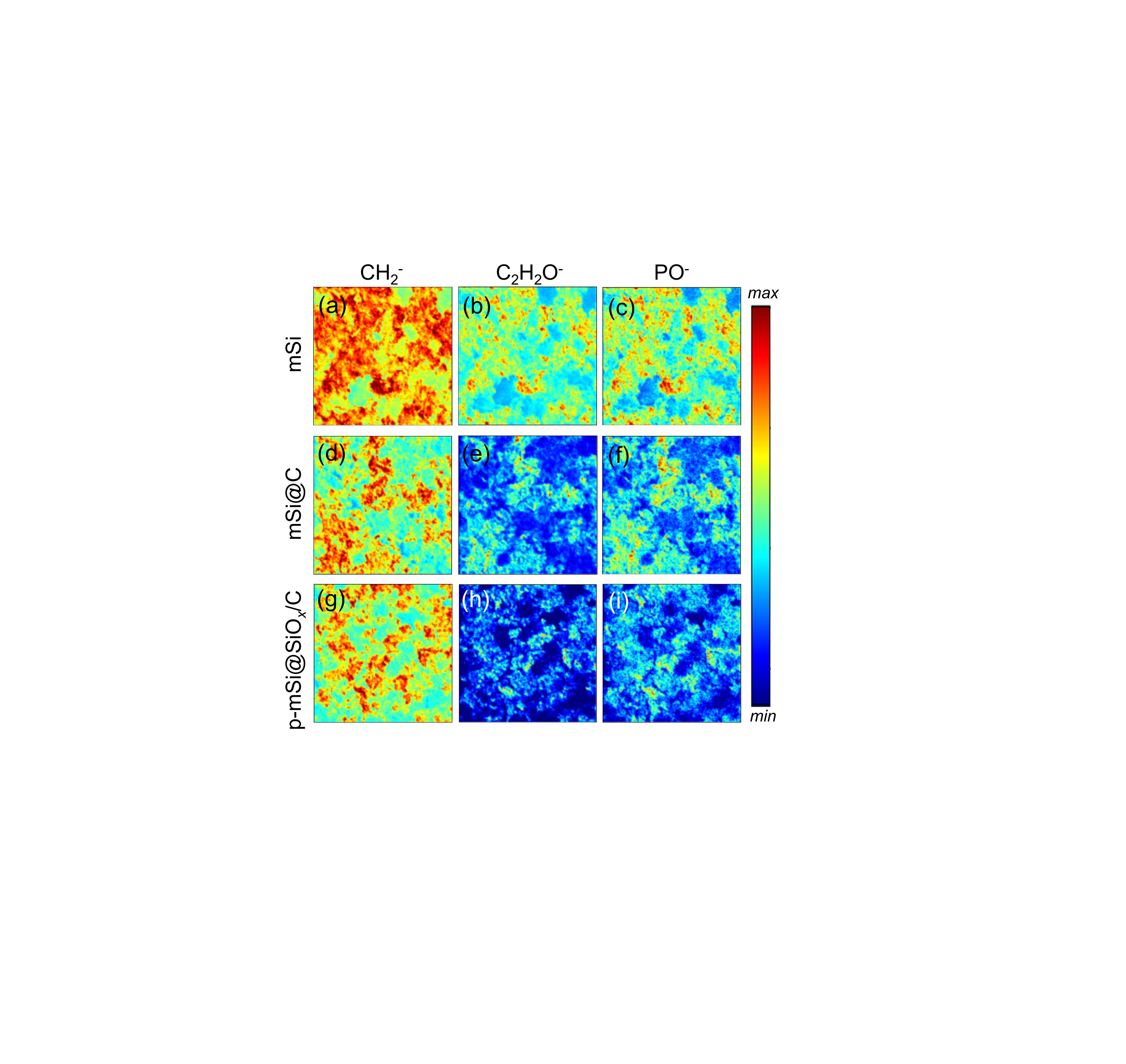


**Fig. S27** Surface TOF-SIMS mapping images of (**a, d, g**) CH_2_^-^, (**b, e, h**) C_2_H_2_O^-^ and (**c, f, i**) PO^-^ signals for the cycled mSi, mSi@C and p-mSi@SiO*_x_*/C electrodes


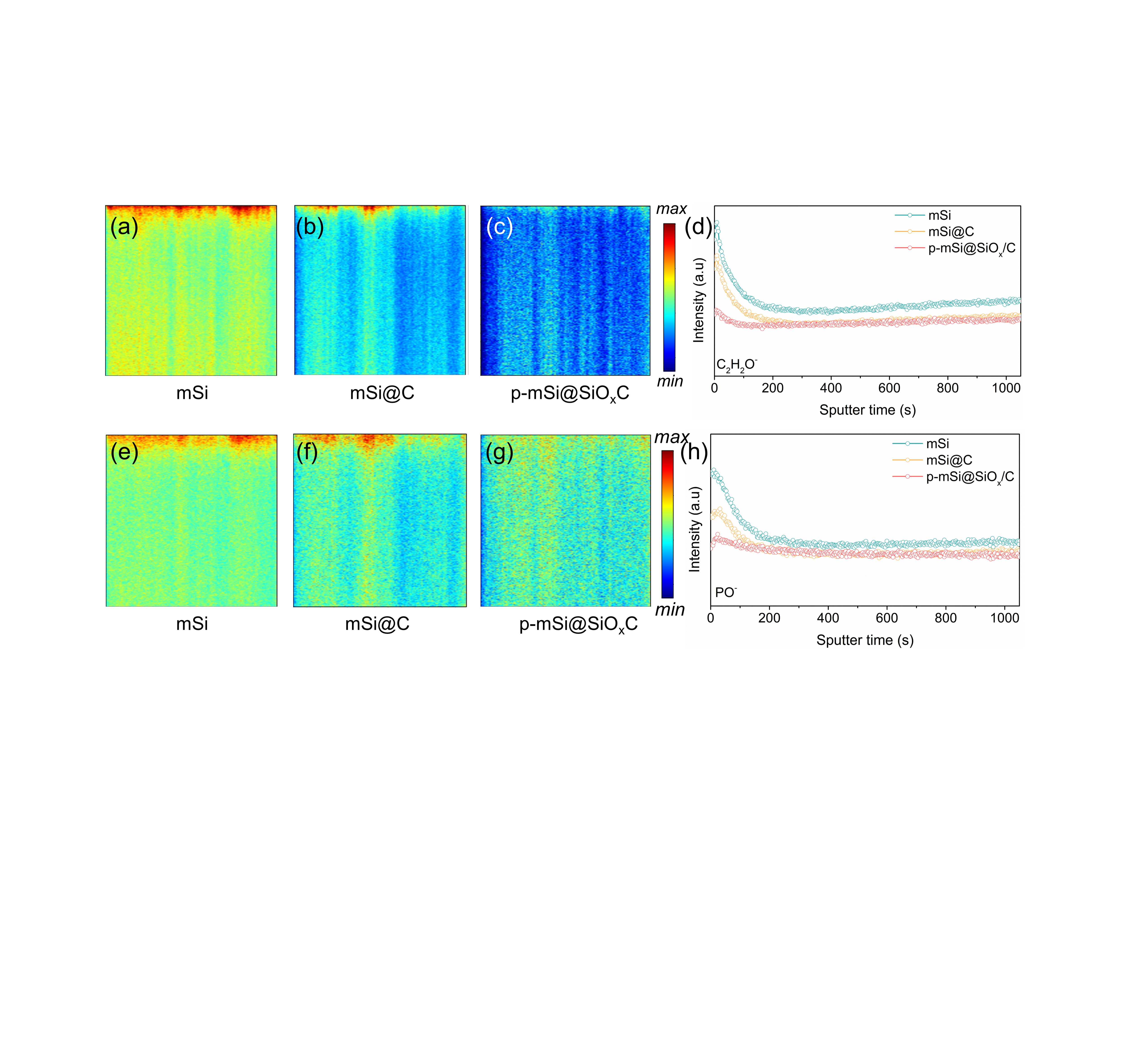


**Fig. S28** In-depth TOF-SIMS mapping images and analysis curves of (**a-d**) C_2_H_2_O^-^ and (**e-h**) PO^-^ signals for the cycled mSi, mSi@C and p-mSi@SiO*_x_*/C electrodes


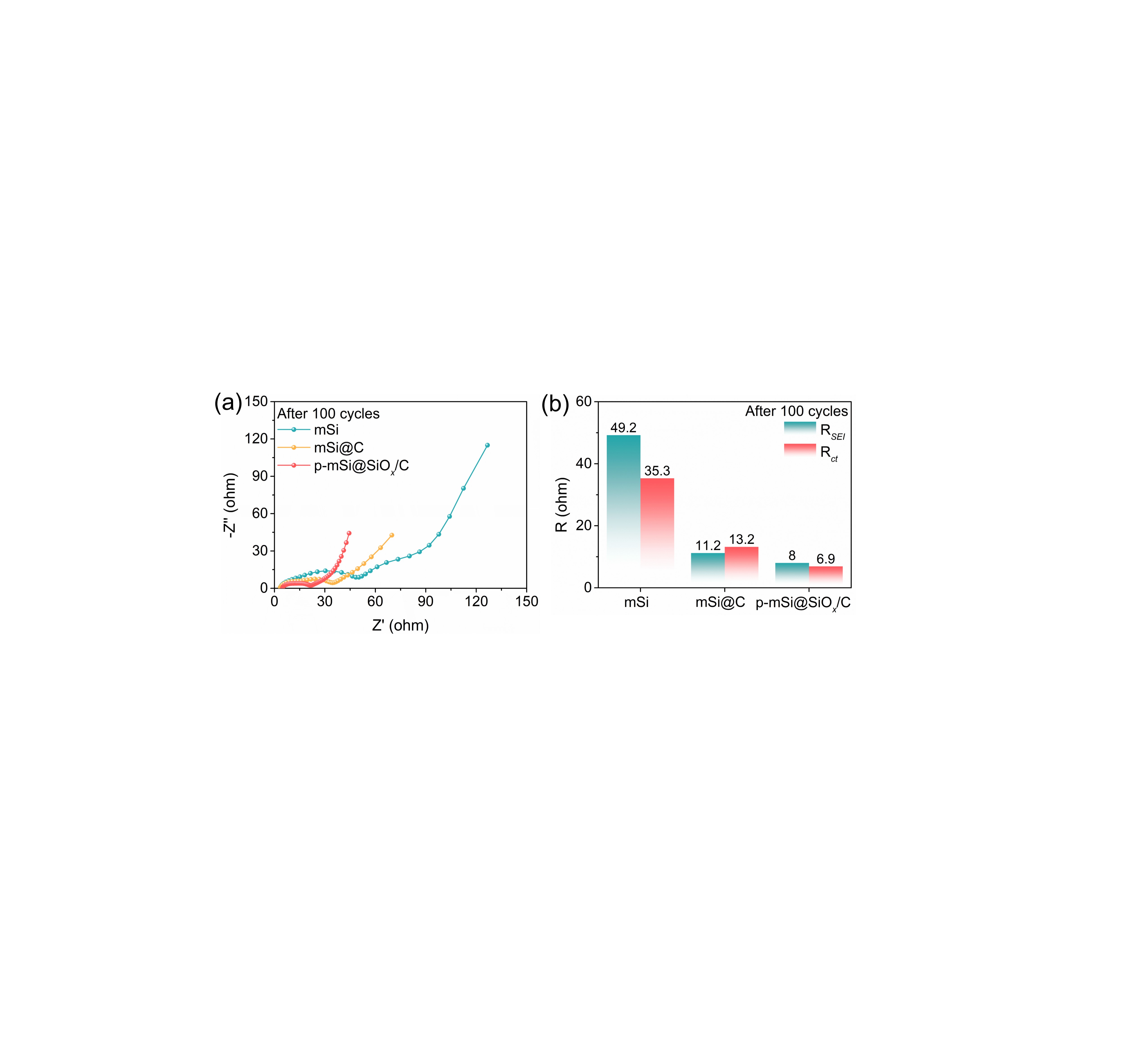


**Fig. S29.** Nyquist plots and corresponding fitted R*_SEI_*/R*_ct_* values for of mSi, mSi@C and p-mSi@SiO*_x_*/C after 100 cycles

**Table S1** The dosage of HEC and AM for the different as-prepared samples

| **Materials** | **HEC (g)** | **AM (g)** |
| --- | --- | --- |
| mSi@C | 0.029 | 0.174 |
| p-mSi@SiO*_x_*/C | 0.04 | 0.24 |
| p-mSi@SiO*_x_*/C-3 | 0.044 | 0.266 |
| p-mSi@SiO*_x_*/C-5 | 0.046 | 0.276 |
| p-mSi@SiO*_x_*/C-7 | 0.048 | 0.289 |

**Table S2** The production cost of single preparation flow based on the 50 g MSi input

| Energy cost | | | Material cost | | | Total cost ($) |
| --- | --- | --- | --- | --- | --- | --- |
| Treatments | electricity consumption (kWh) | Cost ($) | Material (g) | Amount (g) | Cost ($) |  |
| Ball milling | 27.06 | 2.71 | PV Si waste | 50 | 0.27 | 3.44 |
|  |  |  | Li metal | 0.4 | 0.03 |  |
| Heating | 3.2 | 0.32 | HEC | 10 | 0.03 |  |
|  |  |  | AM | 60 | 0.08 |  |

a. Electricity, high voltage {CN} | market group for | $ 0.1/kWh

b. PV Si waste | unknown market price, replaced by the PV Si {CN} | $5.49/kg

c. Li metal, industrial grade, {CN} | market for | $79.63/kg

d. HEC, industrial grade, {CN} | market for | $3.43/kg

e. AM, industrial grade, {CN} | market for | $1.28/kg

**Note S3:**

In our work, the used planetary ball milling process can handle a maximum of 50 g MSi per batch, yielding approximately 70 g of p-mSi@SiO*_x_*/C materials. This throughput poses a challenge for industrial-scale production requiring kilogram-to-hundred-kilogram outputs. Moreover, we have estimated the cost per production batch based on the input of 50 g MSi (yielding about 70 g product). As shown in Table S2, the total cost is about $3.43, suggesting a production cost of $0.05 per 1 g p-mSi@SiO*_x_*/C, which is comparable to the price of commercial Si/C anode. Meantime, it can be observed that the energy consumption of ball milling accounts for more than 78% of total expenses. Therefore, developing more advanced ball milling equipment with enhanced energy efficiency and scaled throughput is of great significance to improve the industrial-scale production potential and economic viability of our developed technology.

**Table S3** Electrochemical characteristics and the calculated energy density of mSi, mSi@C and p-mSi@SiO*_x_*/C at various current densities

| Samples | Current (A g⁻^1^) | Average voltage (V) | | Reversible capacity | | U*_R_*  (Wh L⁻^1^) |
| --- | --- | --- | --- | --- | --- | --- |
|  |  | Lithiation | Delithiation | Gravimetric (mAh g⁻^1^) | Volumetric (Ah L⁻^1^) |  |
| mSi | 0.2 | 0.147 | 0.375 | 1894.4 | 2706.3 | 1009.3 |
|  | 0.5 | 0.127 | 0.371 | 1609.2 | 2298.9 | 990.0 |
|  | 1 | 0.103 | 0.374 | 1366.6 | 1952.3 | 966.2 |
|  | 2 | 0.109 | 0.391 | 1117.24 | 1596.1 | 929.5 |
|  | 5 | 0.084 | 0.489 | 697.0 | 995.7 | 813.9 |
| mSi@C | 0.2 | 0.193 | 0.466 | 1702.7 | 2541.3 | 975.9 |
|  | 0.5 | 0.191 | 0.456 | 1607 | 2398.5 | 971.6 |
|  | 1 | 0.193 | 0.461 | 1462.5 | 2182.8 | 958.0 |
|  | 2 | 0.189 | 0.500 | 1274.9 | 1902.8 | 927.9 |
|  | 5 | 0.17 | 0.594 | 920.4 | 1373.7 | 850.6 |
| p-mSi@SiO*_x_*/C | 0.2 | 0.194 | 0.464 | 1783.6 | 2744.0 | 985.4 |
|  | 0.5 | 0.193 | 0.448 | 1772.3 | 2726.6 | 989.3 |
|  | 1 | 0.194 | 0.449 | 1653.1 | 2543.2 | 980.8 |
|  | 2 | 0.190 | 0.482 | 1474.6 | 2268.6 | 957.2 |
|  | 5 | 0.177 | 0.563 | 1123.0 | 1727.7 | 896.4 |

**Table S4** Comparison of p-mSi@SiO*_x_*/C with the previous micro-Si based anode

| No. | Materials | Current density (A g^-1^) | Cycle number | Capacity (mAh g^-1^) | Refs. |
| --- | --- | --- | --- | --- | --- |
| 1 | Si-NHC@CNFs | 1 | 100 | 928.4 | [S1] |
| 2 | p-Si@SiO_x_/Ag/CN | 1 | 50 | 830.6 | [S2] |
| 3 | Si/AC@GC-700 | 1 | 200 | 956 | [S3] |
| 4 | SiFe | 0.5 | 200 | 803 | [S4] |
| 5 | p-SiO_x_@0.3TiO_2_@C | 0.7 | 500 | 805.9 | [S5] |
| 6 | SiO@LCO | 0.2 | 300 | 588.2 | [S6] |
| 7 | μHGC+nHGC | 0.8 | 500 | 804 | [S7] |
| 8 | Si/SiO_x_-ZnO-C | 0.5 | 200 | 633 | [S8] |
| 9 | Si/C-650@C-600 | 1 | 300 | 846 | [S9] |
| 10 | Si@SiO_x_/WCGCs | 0.1 | 100 | 869.88 | [S10] |
| 11 | Si/Ag/C | 1 | 300 | 706 | [S11] |
| 12 | μP-Si@CNT@C | 0.5 | 200 | 1000 | [S12] |
| 13 | DE-0.5 | 1 | 200 | 444 | [S13] |
| 14 | pSi/MXene | 1 | 500 | 555 | [S14] |
| 15 | DP-Si-1.0 | 1 | 300 | 587.2 | [S15] |
| 16 | PoHC/Si@C | 1.5 | 200 | 550 | [S16] |
| 17 | Si/CoMo@NCP | 1 | 400 | 745 | [S17] |
| 18 | M-Si@SiO*_x_*@C | 1.2 | 200 | 886 | [S18] |
| 19 | Si@8Z | 1 | 650 | 818.5 | [S19] |
| 20 | D-Si@RF-CTP | 0.5 | 60 | 900.8 | [S20] |
| 21 | Si@C@CNTs | 0.5 | 500 | 968 | [S21] |
| 22 | SHT-3@PC@SnS_2_ | 1 | 200 | 1125.3 | [S22] |
| 23 | DP Si/SiO_x_@Li–C | 0.1 | 100 | 809.8 | [S23] |
| 24 | PSi/TiN@NC | 0.5 | 300 | 923.6 | [S24] |
| 25 | V–SiO*_x_*@AP@C | 1 | 200 | 761.5 | [S25] |
|  | p-mSi@SiO*_x_*/C | 1 | 500 | 901.1 | **This work** |

**Supplementary References**

1. D.-C. Jia, Y.-Y. Feng, C.-L. Zhang, J.-J. Li, B.-W. Zhang et al., Freestanding carbon fiber-confined yolk-shelled silicon-based anode for promoted lithium storage applications. Rare Met. **42**(11), 3718–3728 (2023). <https://doi.org/10.1007/s12598-023-02380-3>
2. Y. Li, G. Chen, H. Yang, X. Geng, Z. Luo et al., Three-dimensional porous Si@SiOx/Ag/CN anode derived from deposition silicon waste toward high-performance Li-ion batteries. ACS Appl. Mater. Interfaces **15**(37), 43887–43898 (2023). <https://doi.org/10.1021/acsami.3c09561>
3. S.H. Yang, J.K. Kim, D.-S. Jung, Y.C. Kang, Facile fabrication of Si-embedded amorphous carbon@graphitic carbon composite microspheres *via* spray drying as high-performance lithium-ion battery anodes. Appl. Surf. Sci. **606**, 154799 (2022). <https://doi.org/10.1016/j.apsusc.2022.154799>
4. M. Li, J. Qiu, S. Zhang, P. Zhao, Z. Jin et al., Micrometer-sized ferrosilicon composites wrapped with multi-layered carbon nanosheets as industrialized anodes for high energy lithium-ion batteries. J. Energy Chem. **50**, 286–295 (2020). <https://doi.org/10.1016/j.jechem.2020.03.077>
5. F. Dou, Y. Weng, G. Chen, L. Shi, H. Liu et al., Volume expansion restriction effects of thick TiO_2_/C hybrid coatings on micro-sized SiO*_x_* anode materials. Chem. Eng. J. **387**, 124106 (2020). <https://doi.org/10.1016/j.cej.2020.124106>
6. Y. Qian, Y. Liang, W. Zhang, B. Xi, N. Lin, Thermal polymerization of ion-modified carbon dots into multi-functional LiF-carbon interface for stabilizing SiO anode. Energy Storage Mater. **63**, 102996 (2023). <https://doi.org/10.1016/j.ensm.2023.102996>
7. Q. Shi, Y. Cheng, J. Wang, J. Zhou, H.Q. Ta et al., Strain regulating and kinetics accelerating of micro-sized silicon anodes *via* dual-size hollow graphitic carbons conductive additives. Small **19**(4), 2205284 (2023). <https://doi.org/10.1002/smll.202205284>
8. Y. Li, G. Chen, H. Wu, H. Ding, C. zhang et al., Constructing triple-protected Si/SiOx@ZnO@C anode derived from volatile silicon waste for enhanced lithium storage capacity. Appl. Surf. Sci. **634**, 157651 (2023). <https://doi.org/10.1016/j.apsusc.2023.157651>
9. J. Wan, H. Dong, Z. Zhao, H. Wang, H. Xie et al., Lowering oxygen content on the surface of Si/C composite anode material of lithium-ion batteries with calcium carbide. J. Energy Storage **70**, 107913 (2023). <https://doi.org/10.1016/j.est.2023.107913>
10. S.-F. Liu, C.-H. Kuo, C.-C. Lin, H.-Y. Lin, C.-Z. Lu et al., Biowaste-derived Si@SiOx/C anodes for sustainable lithium-ion batteries. Electrochim. Acta **403**, 139580 (2022). <https://doi.org/10.1016/j.electacta.2021.139580>
11. Y. Li, G. Chen, W. Liu, C. zhang, L. Huang et al., Construction of porous Si/Ag@C anode for lithium-ion battery by recycling volatile deposition waste derived from refining silicon. Waste Manag. **156**, 22–32 (2023). <https://doi.org/10.1016/j.wasman.2022.11.022>
12. L. Sun, Y. Liu, L. Wang, Z. Chen, Z. Jin, Stabilizing porous micro-sized silicon anodes *via* construction of tough composite interface networks for high-energy-density lithium-ion batteries. Nano Res. **17**(11), 9737–9745 (2024). <https://doi.org/10.1007/s12274-024-6937-2>
13. H. Ruan, S. Guo, L. Zhang, Y. Liu, L. Li et al., Boosting lithium storage performance of diatomite derived Si/SiOx micronplates *via* rationally regulating the composition, morphology and crystalline structure. Ceram. Int. **48**(12), 17510–17517 (2022). <https://doi.org/10.1016/j.ceramint.2022.03.015>
14. Z. Zhang, H. Ying, P. Huang, S. Zhang, Z. Zhang et al., Porous Si decorated on MXene as free-standing anodes for lithium-ion batteries with enhanced diffusion properties and mechanical stability. Chem. Eng. J. **451**, 138785 (2023). <https://doi.org/10.1016/j.cej.2022.138785>
15. P. Duan, R. Zhang, Z. Wu, Z. Yang, H. Li et al., Design and construction of porous silicon materials as stable anodes for lithium-ion batteries. Ind. Eng. Chem. Res. **62**(18), 6995–7006 (2023). <https://doi.org/10.1021/acs.iecr.3c00084>
16. S. Yi, Z. Yan, X. Li, Z. Wang, P. Ning et al., Design of phosphorus-doped porous hard carbon/Si anode with enhanced Li-ion kinetics for high-energy and high-power Li-ion batteries. Chem. Eng. J. **473**, 145161 (2023). <https://doi.org/10.1016/j.cej.2023.145161>
17. H. Ruan, L. Zhang, S. Li, K. Wang, W. Huang et al., Carbon polyhedra encapsulated Si derived from Co-Mo bimetal MOFs as anode materials for lithium-ion batteries. J. Mater. Sci. Technol. **159**, 91–98 (2023). <https://doi.org/10.1016/j.jmst.2023.03.009>
18. T. Meng, B. Li, C. Liu, Q. Wang, D. Shu et al., Molecular cooking: Amino acids trap silicon in carbon matrix to boost lithium-ion storage. Energy Storage Mater. **46**, 344–351 (2022). <https://doi.org/10.1016/j.ensm.2022.01.005>
19. D. Zhang, R. Yang, J. Zhou, W. Liu, H. Qin et al., Uniform Li-ion diffusion and robust solid electrolyte interface construction for kilogram-scale Si@ZIF powder as the anode in Li-ion batteries. Energy Storage Mater. **63**, 102976 (2023). <https://doi.org/10.1016/j.ensm.2023.102976>
20. H. Li, Z. Chen, Z. Kang, W. Liu, Y. Chen, High-density crack-resistant Si-C microparticles for lithium ion batteries. Energy Storage Mater. **56**, 40–49 (2023). <https://doi.org/10.1016/j.ensm.2022.12.045>
21. X. Fan, T. Cai, S. Wang, Z. Yang, W. Zhang, Carbon nanotube-reinforced dual carbon stress-buffering for highly stable silicon anode material in lithium-ion battery. Small **19**(30), 2300431 (2023). <https://doi.org/10.1002/smll.202300431>
22. X. Du, Y. Huang, Z. Zhou, C. Chen, Constructing yolk@multi-shell free-standing anodes with porous carbon tube and SnS_2_ nanosheets for Si-based lithium-ion batteries. J. Mater. Sci. Technol. **220**, 23–29 (2025). <https://doi.org/10.1016/j.jmst.2024.09.018>
23. Y. Zhu, H. Zhang, X. Zeng, T. Han, M. Qi et al., Engineering a dual-porous Li-rich carbon-coated Si/SiOx nanosphere as a high-performance Li-ion battery anode. Chem. Commun. **61**(17), 3564–3567 (2025). <https://doi.org/10.1039/D4CC06590E>
24. Y. Li, L. Pang, Y. Li, Z. Li, P. Xiao, Dramatic enhancement enabled by introducing TiN into bread-like porous Si-carbon anodes for high-performance and safe lithium storage. ACS Appl. Mater. Interfaces. (2024). <https://doi.org/10.1021/acsami.4c11332>
25. J. Luan, H. Yuan, J. Liu, N. Zhao, W. Hu et al., Amorphous AlPO_4_ layer coating vacuum thermal reduced SiO*_x_* with fine silicon grains to enhance the anode stability. Adv. Sci. **11**(36), 2405116 (2024). <https://doi.org/10.1002/advs.202405116>
